# PROFIS: Design of Target-Focused Libraries by Probing Continuous Fingerprint Space with Recurrent Neural Networks

**DOI:** 10.1021/acs.jcim.5c00698

**Published:** 2025-04-28

**Authors:** Hubert Rybka, Tomasz Danel, Sabina Podlewska

**Affiliations:** † Doctoral School of Exact and Natural Sciences, Jagiellonian University, Łojasiewicza 11, 30-348 Kraków, Poland; ‡ Faculty of Chemistry, Jagiellonian University, Gronostajowa 2, 30-387 Kraków, Poland; § Maj Institute of Pharmacology, Polish Academy of Sciences, Smȩtna 12, 31-343 Kraków, Poland

## Abstract

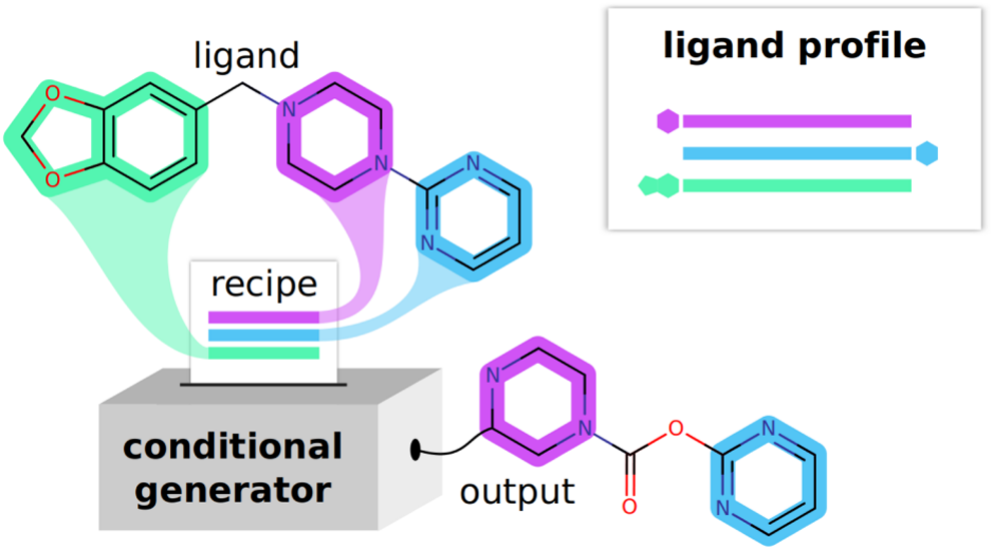

This study introduces PROFIS, a new generative model capable of the design of structurally novel and target-focused compound libraries. The model relies on a recurrent neural network that was trained to decode embedded molecular fingerprints into SMILES strings. To identify potential novel ligands, a biological activity predictor is first trained on the low-dimensional fingerprint embedding space, enabling the identification of high-activity subspaces for a given drug target. The search for latent representations that are expected to yield active structures upon decoding to SMILES is conducted with a Bayesian optimization algorithm. We present the rationale for using SMILES as the output notation of the recurrent neural network and compare its performance with models trained to decode DeepSMILES and SELFIES strings. The paper demonstrates the application of this protocol to generate candidate ligands of the dopamine D_2_ receptor. It also emphasizes the effectiveness of our approach in scaffold-hopping, which is valuable for designing ligands outside the already explored chemical space. We present how passing engineered molecular fingerprints through PROFIS network can be utilized to generate diverse libraries of analogs for a drug molecule of choice. It is worth noting that the protocol is versatile and it can be employed for any biological target, given the availability of a dataset containing known ligands. The potential for widespread use of PROFIS is secured by scripts shared by the authors on GitHub.

## 1 Introduction

The contemporary landscape of drug discovery is characterized by the increasing complexity of the tasks, the rising cost of research and development, and the demand for faster and more efficient ways to bring innovative therapeutics to market.^1^ As a solution to these challenges, computational methods have become more prevalent to accelerate and streamline the drug design process.^2,3^ Significant advancements in the field of machine learning (ML) have led to a revolution in the pharmaceutical industry, paving the way to faster and more effective drug discovery.^4,5^

Before any computational algorithm can process a molecular structure, it must be encoded in a way that allows the machine to parse it effectively. Arguably, the most easily discernible of those representations are textual sequential encodings of chemical structures that constitute a cornerstone of chemistry-related data science and computing. Several methods have emerged in this domain, including notable approaches such as the Simplified Molecular Input Line Entry System (SMILES),^6^ its ML-suited counterpart DeepSMILES,^7^ and Self-referencing Embedded Strings (SELFIES^8^).

SMILES, known for its widespread acceptance and simplicity, provides a human-readable representation of molecular structures through linear character strings. While it is widely used by humans, SMILES can pose challenges for computer interpretation. The complex ring closure and branching syntax, based on matched parentheses spread across the string, may hinder the ability of a recurrent neural network (RNN) to learn the underlying rules. Additionally, most organic molecules can have multiple valid SMILES strings depending on how the atoms were numbered before conversion. To address the need for an unambiguous method of deriving SMILES encodings, one can turn to canonical SMILES, ensuring that each chemical structure is assigned exactly one unique canonical SMILES string.^9^

DeepSMILES addresses the challenges associated with SMILES by enhancing the robustness and consistency of molecular representations.^7^ It brings about notable syntactical improvements, including the use of only one ring closure symbol for each atom ring (in contrast to SMILES’ matched ring closure symbols) and the elimination of paired parentheses to designate branches. DeepSMILES was introduced to improve the validity of sequences produced by generative models based on deep neural networks.

Another textual representation, which is claimed to be less susceptible to ambiguous syntax, is SELFIES.^8^ What distinguishes SELFIES from other encodings is its unique robustness in comparison to other encodings, such as SMILES and DeepSMILES, as every combination of SELFIES tokens necessarily represents a valid molecule.

**Figure 1:**
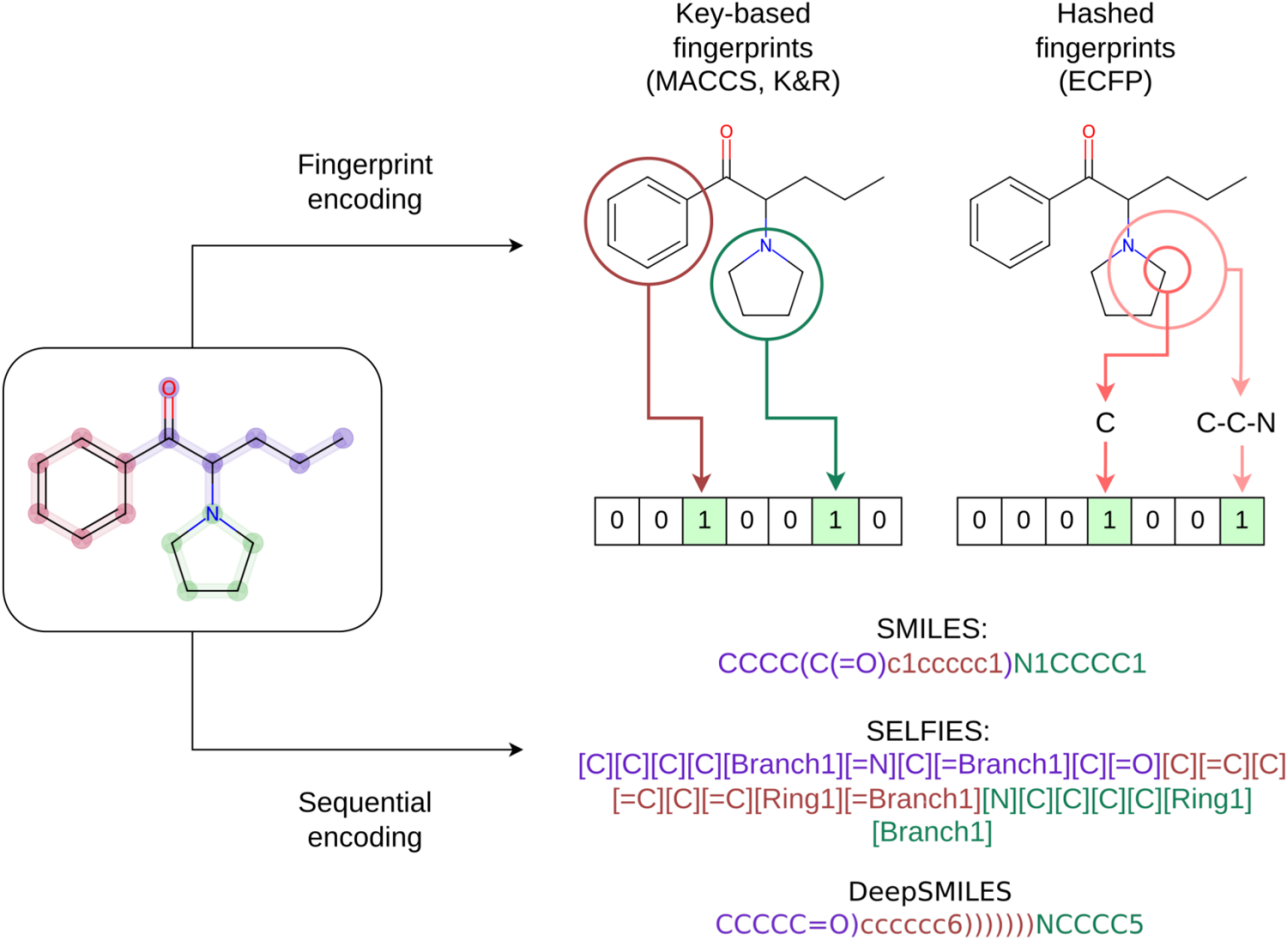
Different approaches to encoding a chemical structure into a machine-readable format.

Molecular fingerprints (FPs) are structural representations of chemical compounds in the form of binary or numerical vectors, capturing critical information about a molecule’s constituent atoms, bonds, and substructures. In the context of ML and drug design, some of the commonly employed FPs are Extended-Connectivity Fingerprints (ECFP),^10^ MACCS keys^11^ or Klekota-Roth fingerprints (KRFP).^12^ In contrast to molecular graphs or textual encodings, FPs have the potential to extract information about biochemically relevant functional groups and present it in a compact, machine-readable format. Circular FPs, such as ECFP, represent molecular structures by encoding circular atom neighborhoods. The significance of subsequent bits is not predefined; thus, they can encode a variety of molecular structures. Key-based FPs, such as MACCS or KRFP, follow a set of substructural keys and are constructed by identifying the presence or absence of certain structural motifs in the molecule. The comparison of sequential and FP-based encoding methods is presented in Figure 1. The main disadvantage of molecular FPs is related to difficulties in reconstructing the encoded molecule as the direct information about the connections between particular substructures is lost.

Although still the most popular procedure in the broadly understood computer-aided drug design (CADD) is virtual screening (VS),^13,14^ the popularity of the types of libraries that undergo VS-based evaluation has varied a lot in recent years. Usually, commercially available libraries are the first choice when searching for new potential ligands; however, especially in the case of highly explored targets, they had already been screened by many groups using a great variety of tools. As an alternative, completely novel sets of compounds have been examined as potential sources of new ligands. The compound structures are constructed based on already-known ligands using approaches such as scaffold-hopping, ligand hybridization, or bioisosteric replacement. Alternatively, the specific ML subfield of generative approaches can be used, examples of which are summarized below.

One of the most popular generative approaches in CADD is variational autoencoder (VAE),^15^ which has already proven to be invaluable in chemistry for its ability to create structured, continuous latent space representations of molecular structures.^16,17^ These latent representations capture essential chemical features and facilitate the generation and optimization of new molecules. When integrated with RNNs, which are particularly well-suited for sequential molecule encodings, the resulting models excel in learning and generating molecular structures with high fidelity.^18,19^

The first notable achievement in the development of a SMILES-based autoencoder model is attributed to Gómez-Bombarelli et al.^16^ The group constructed a VAE architecture that combines a one-dimensional convolutional encoder with an RNN decoder. The latent space of this VAE model provides a continuous representation of molecular structure, enabling the application of gradient-based search techniques to discover optimized functional compounds.

Despite the challenges associated with reconstructing a molecule from its molecular FP, various research groups have made significant progress using this representation in the context of VAEs. Lovrić et al.^20^ showed that the latent space of a trained VAE provides ground for further ML classification tasks, which can outperform classification on uncompressed FPs. While successfully employed in property prediction tasks,^21,22^ molecular FPs rarely find use in generative models, as the transformation from FPs to molecular graphs is usually nontrivial. Some attempts have been made to reverse-engineer FPs into explicit structures, with notable examples including Neuraldecipher,^23^ a model designed to deduce molecular structures from ECFPs. Recently, Ucak et al.^24^ utilized a transformer model to retrieve SMILES and SELFIES strings from various types of molecular FPs, including key-based ones. An approach to FP utilization in a generative model has been made by Kotsias et al.,^25^ who constructed a conditional RNN that can be seeded with FPs or other molecular descriptors to obtain compound libraries that exhibit properties associated with the provided seed.

In this study, we propose a novel generative model, PROFIS, which allows for the design of target-focused compound libraries by probing continuous fingerprint space with RNNs. PROFIS is an innovative VAE that encodes molecular FPs and decodes molecular structures in a sequential notation while ensuring alignment with the initial FP description. We evaluated the performance of our architecture on two types of FPs: KRFP^12^ and circular extended-connectivity fingerprints (ECFP4).^10^ Furthermore, we have thoroughly examined the implications of utilizing SMILES,^6^ DeepSMILES,^7^ and SELFIES^8^ as the output encoding format of the RNN.

In the task of generating potential novel ligands, PROFIS employs a Bayesian search algorithm in tandem with a QSAR model to traverse the space of embedded molecular FPs in search of subspaces that correspond to potential good binders. The latent vectors sampled from those subspaces are then decoded into textual formats such as SMILES or DeepSMILES using a recurrent neural network. Since many FPs do not determine the full chemical structure, our method can generate diverse molecules that match the particular FP description. The generated structures are target-specific, which allows for the generation of potential ligands tailored to a specific receptor. We prove that PROFIS exhibits excellent scaffold-hopping capabilities, enabling the exploration of novel chemical space, an essential feature of computational tools for de novo ligand generation. In the study, we present the application of our protocol in the task of ligand generation for the dopamine D_2_ receptor (D_2_R); however, the developed methodology is universal and can be applied to any biological target, provided a dataset of known ligands is available. To facilitate the widespread use of PROFIS, we share all of the scripts needed to run the developed protocol via GitHub.

## 2 Methods

### 2.1 Recurrent Neural Network

PROFIS relies on an RNN that maps the space of embedded molecular fingerprints onto sequential notations of the molecular structure. A three-layer gated recurrent unit (GRU)^26^ is used in our experiments (following Yang et al.^27^ where similar performances of GRU and LSTM were reported, we decided to use just GRU in our studies). Each molecule is represented as a pair (*x̅*, *s*), where  is a *d*-dimensional encoding of the chemical structure (e.g., a molecular FP), and *s* = (*s*_1_, *s*_2_, ···, *s*_*k*_) is the sequential representation of this compound consisting of tokens *s*_*i*_ ∈ *T*, *i* ∈ {1, 2, ···, *k*}. We used 29 (SMILES), 28 (SELFIES), or 46 (DeepSMILES) structure tokens. The list of tokens selected for the task is presented in Table S4. The sequence length *k* is set to 100, and shorter strings are padded by the use of a padding token.

The GRU generator with *N* layers, where the *n*th layer is denoted , is used to map embedded molecular fingerprints onto sequences as follows
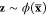
1
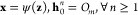
2
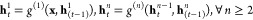
3
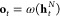
4where  is a stochastic structure encoder described in the following section,  is a multilayer perception (MLP) to map the latent space to the GRU input, and  is an MLP with a softmax layer at the end used to transform GRU outputs into token probabilities.

**Figure 2:**
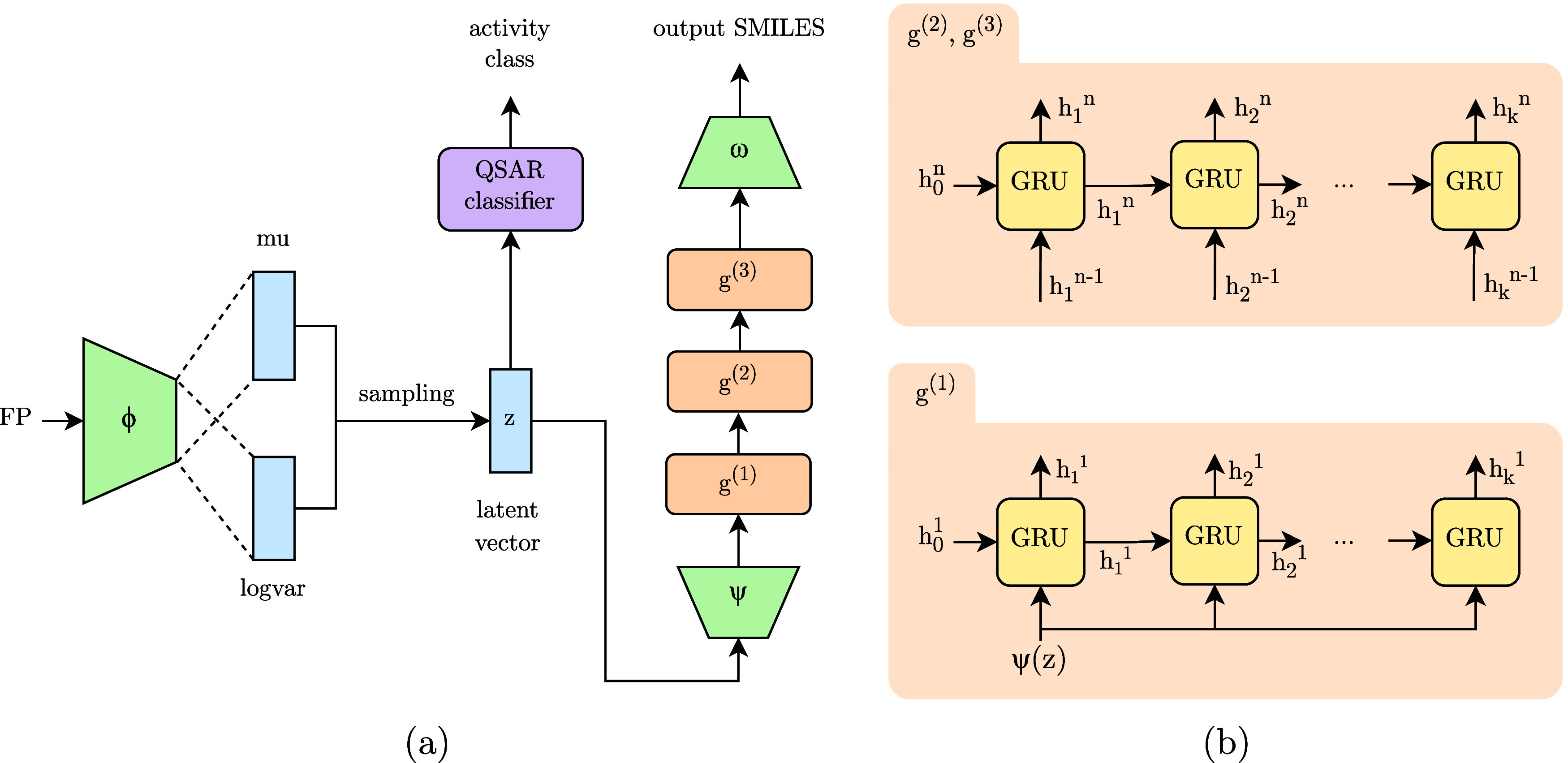
(a) Architecture of PROFIS. In training, FPs are used as an input to the encoder ϕ to sample a latent vector *z*, from which a three-layer GRU network *g* decodes molecules as a sequence of tokens. Additionally, a QSAR classifier is trained on top of the latent space to enable the identification of potent chemical subspaces. (b) Detailed look into the implementation of our three-layer GRU decoder. Hidden state *h*_0_ of each GRU layer is initialized as a zero tensor.

The whole network is trained with a two-factor loss function, similar in principle to the VAE cost function. Hyperparameters were tuned using a Bayesian optimization strategy (see Table S2). In our experiments, the network ϕ is a two-layer fully connected network with a ReLU activation function, ω and ψ are one-layer networks, and the GRU internal hidden dimension *m* is set to 512. The whole network is trained using the Adam optimizer. The overall architecture is presented in Figure 2.

### 2.2 Stochastic Structure Encoder

We propose a stochastic structure encoder ϕ that can produce multiple structure representations given the input vector **x̅** encoding the chemical structure. The encoder network consists of two stacked fully connected layers of size (1024, 1024), and two parallel output layers of size 32. These two latent vectors are then used as an input to the reparametrization trick,^15^ as it combines them into a single (latent) vector of size 32. Due to the nature of the reparametrization trick, this encoding process is fully differentiable so that it can be trained along with the GRU generator.

### 2.3 Training Dataset

The RNN training dataset comprised molecules obtained from the ChEMBL32^28^ and ZINC^29^ databases. The ChEMBL database referring to the human D_2_R was filtered for “Small Molecules”, with molecular weights falling within the range of 200–450 g/mol and exhibiting no violations of Lipinski’s rule of five. The ZINC-250k subset of the ZINC database was used without any filters applied. Charged atoms were neutralized and the counterions were removed using the Uncharger functionality of rdkit.Chem.MolStandardize module.^30^ All stereochemistry information about the molecules was disregarded. KRFP and ECFP4 representations were generated for each of the preprocessed molecules. These operations were executed with the RDKit Python library.^30^ The final data set consisted of 1,126,085 compounds encoded in the canonical SMILES notation.

The dataset was divided into training and test sets, in the ratio of 9:1, in the following manner: first, all of the compounds are grouped according to their Bemis–Murcko scaffold.^31^ Next, a random group of compounds is selected and appended to the training set. If the size of the resulting training set is larger than 90% of the original dataset, the group is appended to the testing set instead. This continued until all scaffold groups are assigned.

### 2.4 Objective Function

The loss function optimized in the course of training can be formally defined as
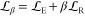
5where  is the error of sequential encoding reconstruction given the corresponding molecular FP (here termed “reconstruction error”) and  is a KL regularization term.

We observed that the value of β = 0.1 resulted in a reasonable balance between the latent space regularization and reconstruction capacity of the model and used it throughout our experiments. The reconstruction error  is calculated as a cross-entropy loss on the token logits generated by the RNN decoder and the sequential representation of compounds, which were either plain SMILES or representations derived from canonical SMILES using selfies^8^ and deepsmiles^7^ Python libraries. The KL loss  is defined as in the standard VAE.^15^

### 2.5 Latent Space Regularization Annealing

During the initial stages of training, as the encoder has not yet learned a meaningful representation of the training data, the autoregressive decoder may become misled by the ineffectuality of latent encodings and learn to ignore that information entirely. The consequence of such behavior is known as the Kullback–Leibler (KL)-vanishing problem,^32^ a position in which the optimizer quickly minimizes the Kullback–Leibler divergence (KLD) regularization term of the VAE loss function, while the reconstruction error does not decrease significantly. This results in a highly organized latent space but poor reconstruction efficiency of the trained model and will negatively impact the overall performance of the autoencoder.

To mitigate this effect, we employed a technique known as KLD annealing. This approach is successfully used in many natural language processing tasks and relies on the fact that the relative weight of the KLD term may be changed during the training. We observed that implementing a cosine annealing schedule during the first 50 epochs of training helped to significantly reduce the KL-vanishing problem, resulting in a good balance between latent space regularization and the reconstructive capacity of the whole network.

### 2.6 Latent Space QSAR Models

The study involved the generation of potential novel dopamine D_2_R ligands by performing an ML-assisted search on the space of molecular FP embeddings *z*. Enabling this search is a QSAR classifier model developed to discern the subspaces of the latent space that encode active D_2_R ligands. These latent representations were converted into sequential encodings by utilizing our trained RNN decoder.

**Figure 3:**
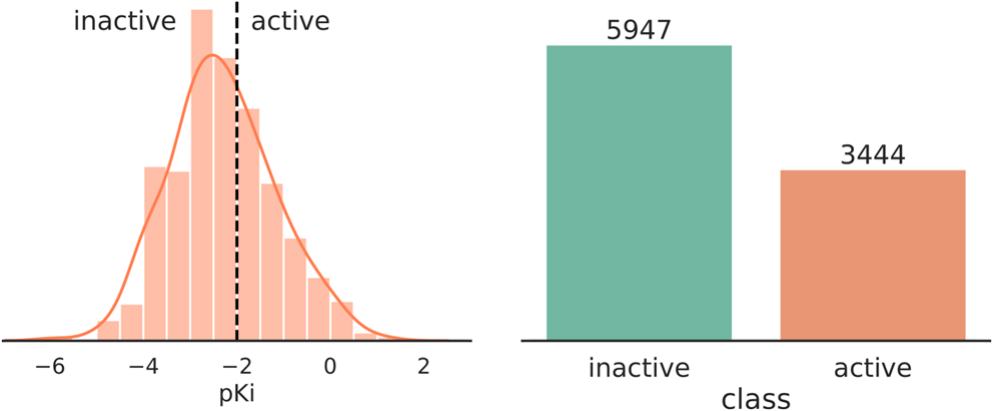
Distribution of p*K*_*i*_ values and the assigned activity classes in the data set of D_2_R ligands.

The classifier was trained using a dataset of D_2_R ligands extracted from the ChEMBL32^28^ database, specifically filtered for human D_2_R ligands, and with associated *K*_*i*_ values less than 10 μM. The distribution of molecular properties for this dataset is presented in Figure S3. Dataset preparation followed the same procedure as that employed for the RNN decoder training set. All molecules with the value of *K*_*i*_ less than or equal to 100 nM were assigned the “active” class, and the others were marked as “inactive”. The distribution of *K*_*i*_ values and assigned activity classes for this dataset is presented in Figure 3. Before training, KRFP and ECFP4 were computed and subsequently encoded into latent vectors using the trained encoder.

Several different QSAR models were developed and evaluated using a nested 5-fold cross-validation (CV) strategy. The classification algorithms included were as follows: random forest (RF), support vector machines (SVM), gradient-boosted decision trees (XGB), and MLP. A detailed description of our CV strategy is presented in the Supporting Information, along with all hyperparameter grids used for model tuning (Table S3). Models were implemented, trained, and evaluated using scikit-learn Python library,^33^ except for the XGB classifier, which was implemented with a XGBoost Python library.^34^

To effectively explore the latent space, a Bayesian optimization algorithm was employed in conjunction with the previously trained classifier. The Python implementation of the Bayesian search by Nogueira^35^ was used and run with the following parameter values: *init_points* = 4, *init_points* = 20. The search bounds were determined based on the distribution of the RNN test set vectors in our model’s latent space, namely, μ ± 2σ on every latent dimension. Additionally, we utilized the package’s capability for applying sequential domain reduction during the search in our experiments.

### 2.7 Molecular Docking

A generated compound library of candidate D_2_R ligands was docked to the crystal structure of the respective receptor (PDBID: 6LUQ).^36^ Additionally, we conducted docking of a sample of 150 drug-like molecules obtained from the ChEMBL32 database.^28^ These molecules were selected based on the same criteria as the RNN training dataset and served as a baseline. We also docked a random sample of 150 active (*K*_*i*_ < 100 nM) D_2_R ligands from the latent classifier’s training set.

In order to demonstrate the target-focused nature of the obtained library, the same sets of compounds were docked to a protein that is both structurally and functionally unrelated to D_2_R—bacterial acetylcholinesterase, ChoE (PDBID: 6UQW).^37^

The protein files were prepared for docking by titrating the residues to pH = 7.4 with the use of ProteinPrepare app.^38^ The docking was carried out in GNINA,^39^ with exhaustiveness = 16, *autobox_add* = 10, and the original 6LUQ or 6UQW ligand molecules passed as *autobox_ligand* parameter. The docking score reported in this study is GNINA’s binding affinity (kcal/mol).

## 3 Results

### 3.1 Latent Space Activity Classifier

The cornerstone of our generative model is a low-dimensional embedding space of molecular FPs, which can be effectively searched for subspaces that represent the structure of potent ligands. To enable this task, a QSAR model in the form of an activity classifier was trained on the latent space. It was tasked with learning to assign an “active” or “inactive” label to the latent representations of molecular structures. The hyperparameter tuning and performance evaluation for the latent space classifiers was carried out with a double-nested, 5-fold CV strategy, and the ROC AUC and accuracy scores for the best models are presented in Table 1. The trained classifiers successfully acquired the capability of identifying D_2_R-active subspaces. The performance of our trained latent classifiers was confronted with the classification of uncompressed FPs as a baseline.

**Table 1:** Performance Metrics of the Trained Latent Classifier Models Compared to the Baseline Models Trained Directly on Fingerprints^a^

		ECFP4		KRFP	
model		ROC AUC	accuracy	ROC AUC	accuracy
latent	SVM	0.878 ± 0.006	0.828 ± 0.007	0.878 ± 0.011	0.824 ± 0.012
	RF	0.761 ± 0.014	0.716 ± 0.010	0.768 ± 0.010	0.710 ± 0.010
	XGB	0.882 ± 0.008	0.819 ± 0.010	0.885 ± 0.010	0.824 ± 0.013
	MLP	0.892 ± 0.008	0.825 ± 0.008	0.887 ± 0.013	0.826 ± 0.016
baseline	SVM	0.906 ± 0.008	0.853 ± 0.010	0.905 ± 0.011	0.844 ± 0.010
	RF	0.797 ± 0.017	0.724 ± 0.010	0.822 ± 0.017	0.732 ± 0.010
	XGB	0.910 ± 0.009	0.838 ± 0.010	0.924 ± 0.010	0.855 ± 0.012
	MLP	0.894 ± 0.011	0.834 ± 0.009	0.911 ± 0.010	0.842 ± 0.020

a The classification was performed on the SMILES-based VAEs’ latent space. The mean and error values reported in the table were calculated based on a 5-fold cross-validation strategy.

Compared to the baseline, both the ECFP4 and KRFP-based latent classifiers performed similarly or only slightly worse despite relying on data that has been compressed into a compact 32-dimensional embedding space. In the task of latent space classification, a simple MLP turned out to perform the best in conjunction with both ECFP4 (ROC AUC = 0.892 ± 0.008) and KRFP-based models (ROC AUC = 0.887 ± 0.013), with XGB and SVM models exhibiting comparable or only slightly worse performance in terms of the ROC AUC and accuracy. There are no significant differences in the performances of classifiers trained on embedded KRFP and ECFP4 fingerprints. RF models fall short in terms of ROC AUC and accuracy on both uncompressed FPs and their latent representations, rendering them unsuitable for the task.

These satisfactory results prove that our trained stochastic encoder successfully embeds molecular FPs into a space of only 32 dimensions without the loss of much valuable chemical information. SVM, XGB, and MLP classifiers, with their optimal hyperparameters listed in Table S3, seem to perform well in latent space activity classification tasks. For further experiments requiring latent space classification, the MLP-based QSAR classifier will be used unless otherwise specified.

### 3.2 Libraries Generation

Using the SMILES/KRFP and SMILES/ECFP4 models, along with an MLP-based latent space probing method, two sets of candidate D_2_R ligands were generated. For each of the two types of FPs, we first identified a collection of 50,000 latent embedding with our Bayesian search strategy. The vectors were then decoded to SMILES strings. The validity of the SMILES strings obtained from each VAE is reported in Table 2. The molecular structures of the raw output from the SMILES/ECFP4 and SMILES/KRFP models are depicted in Figures S1 and S2.

**Table 2:** Output Validity of the Models Trained on Both ECFP4 and KRFP Fingerprints, with SMILES, DeepSMILES, and SELFIES as the Output Format of the RNN^a^

		validity [%]			
output	FP type	holdout test set	prior sampling	posterior sampling	PROFIS
SMILES	ECFP4	39	6.8	5.7	3.9
SMILES	KRFP	33	5.7	3.7	1.7
DeepSMILES	ECFP4	40	19.5	7.2	7.2
DeepSMILES	KRFP	34	7.4	6.4	11.8
SELFIES	ECFP4	100	100	100	100
SELFIES	KRFP	100	100	100	100

a The validity was measured by (a) holdout test set (b) sampling and decoding random vectors from , (c) sampling and decoding random vectors from the aggregated posterior distribution of the training set, approximated by , (d) using Bayesian search to identify representations of candidate D_2_R ligands and decoding them according to the PROFIS protocol.

Although not all of the generated molecules possess drug-like physicochemical properties, and some contain atom arrangements that would raise questions about the molecules’ stability and synthetic accessibility, these can be easily filtered off in a lightweight postprocessing step. The filtering step is implemented as part of the PROFIS code, and the criteria can be customized according to needs. We will use the following simple filters for the generation of final libraries; quantitative estimate of drug-likeness (QED)^40^ > 0.5, computed logP (clog *P*) < 8, and maximum ring size = 7.

**Figure 4:**
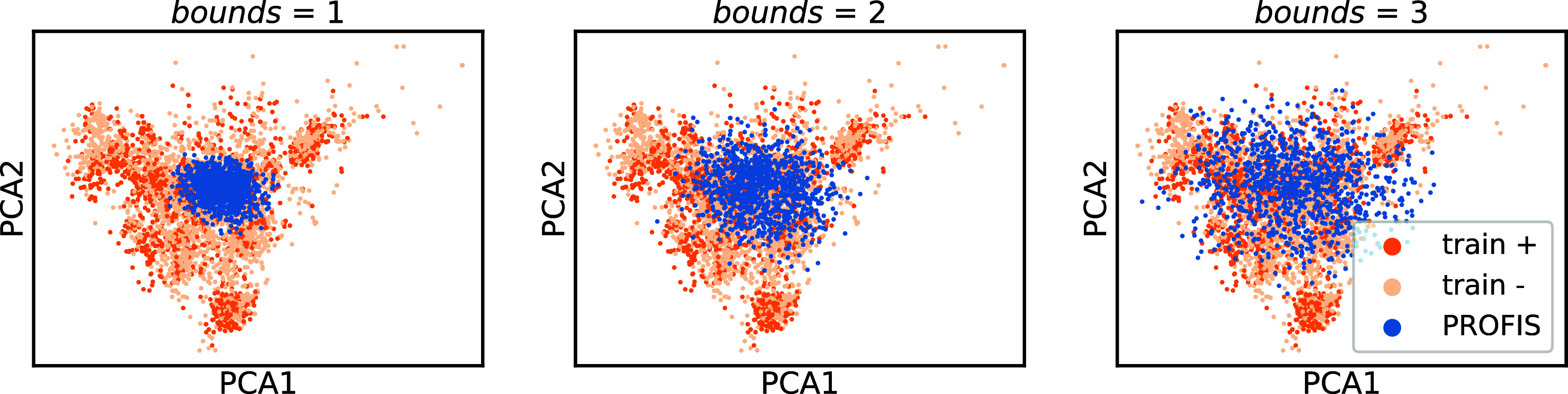
PCA projections of latent encodings of a sample taken from the D_2_R dataset (orange, beige) and latent encodings identified as active by our latent MLP QSAR model (blue). Beige is used to denote poor D_2_R binders (>100 nM) and orange is used to denote good (≤100 nM) D_2_R binders.

**Figure 5:**
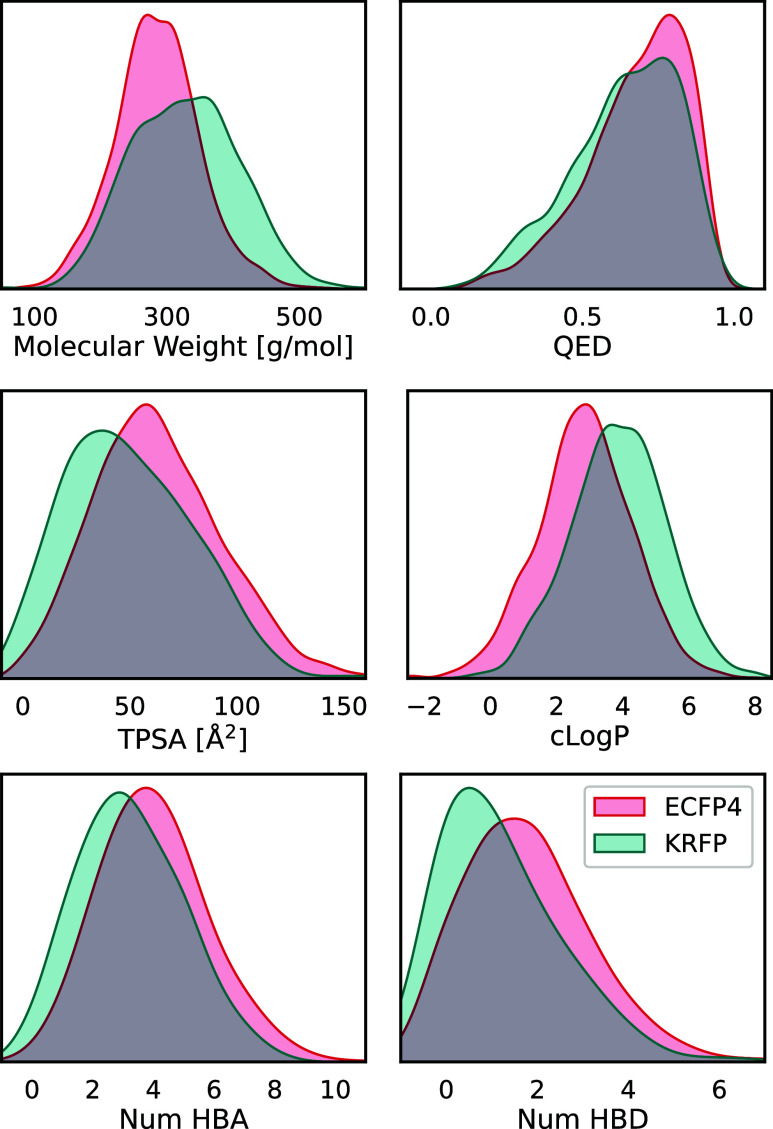
Molecular properties of the compound libraries produced by PROFIS architecture trained on ECFP4 (red) and KRFP (teal) fingerprints: QED,^40^ clog *P*, total polar surface area (TPSA), and the number of hydrogen bond acceptors (HBA) and hydrogen bond donors (HBD).

Models trained on ECFP4 and KRFP fingerprints exhibit differences in the quality and validity of the output molecular structures. PROFIS trained on ECFP4 produces more syntactically correct SMILES strings than its KRFP-based counterpart; however, the output molecules are generally of lower molecular weight (Figure 5), are more hydrophilic, and contain more polar atom groups. It is important to note that the parameter *bounds* of the latent search algorithm greatly influences the quality and validity of output SMILES strings. Increasing the value of *bounds* reduces the mean validity of generated strings, see Table 3, but allows the algorithm to explore broader subspaces of the fingerprint embedding space (see Figure 4), resulting in the final libraries being more diverse in terms of molecular mass and overall structural complexity. The impact of different values of *bounds* parameter on the molecular mass distribution in the generated compound libraries is presented in Figure S4.

**Table 3:** Validity of PROFIS Output SMILES at Different Values of the *Bounds* Parameter^a^

	validity [%] at *bounds*		
model	1.0	2.0	3.0
ECFP4	9.19	3.88	1.81
KRFP	2.93	1.65	0.78

a Validity calculated in an analogous manner as in Table 2.

In this work, we describe compound libraries generated at *bounds* = 2.0, which means that if we were to approximate the fingerprint embedding space with , the Bayesian search algorithm is bounded to the range [μ – 2σ, μ + 2σ] on each latent dimension (Figure 5).

Similarly, two compound libraries were generated using the PROFIS architecture trained to decode DeepSMILES strings (the validity of the generated compounds is summarized in Table 2). As anticipated, the simplified syntax of DeepSMILES led to an increase in the validity of the generated strings compared with those produced by SMILES-based models. The raw output structures from the ECFP4-based DeepSMILES model are provided in Figure S5.

**Figure 6:**
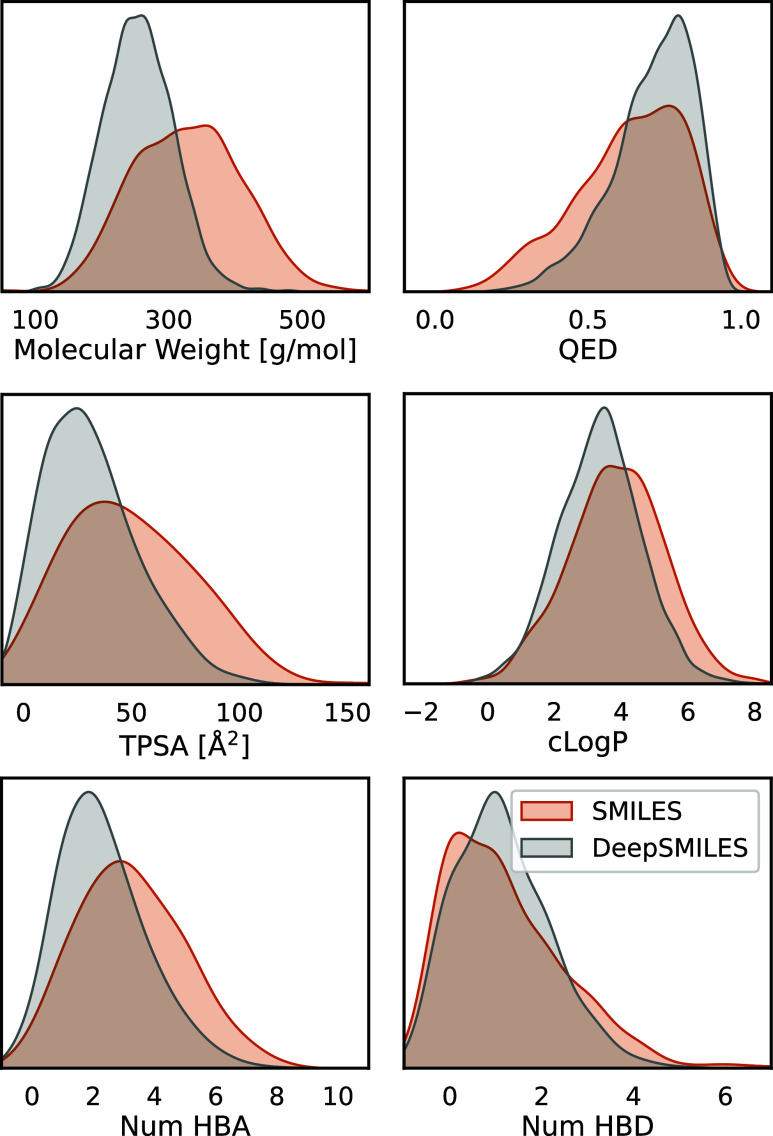
Molecular properties of the compound libraries produced by PROFIS architecture trained on KRFP to decode SMILES (orange) or DeepSMILES (gray) strings: QED,^40^ clog *P*, TPSA, number of HBA, number of HBD.

These libraries were subsequently filtered according to the criteria outlined earlier to draw a comparison between the molecules generated by the SMILES-based and DeepSMILES-based PROFIS models. The distribution of molecular properties for both sets of compounds is illustrated in Figure 6. While the use of DeepSMILES as the output format for the RNN improved the validity of the generated sequences (see Table 2), it resulted in a decrease in the overall quality of the generated chemical structures compared to those generated by SMILES. Specifically, the compounds produced by the DeepSMILES model are lighter and may lack structural complexity, with a population of small (molecular weight between 100 and 200 g/mol) structures not present in the libraries generated with the SMILES-based model.

Prompted by those observations, in this work, we decided to study SMILES-based PROFIS trained on KRFP fingerprints and describe its utility as a generative tool for the design of target-focused compound libraries. The library of molecules filtered with the three simple criteria mentioned above will be utilized in subsequent molecular docking studies, as well as to evaluate the scaffold-hopping potential of PROFIS.

### 3.3 Properties of the Latent Space

The latent space of our model provides a regularized, low-dimensional representation of the structural features. The continuity of structural information in the latent space is outlined by the example of diazepam, a benzodiazepine-class drug, and vectors sampled from the latent space “neighboring” its embedding vector. One can observe gradual changes to the molecular structure as we wander “further” (as measured by euclidean distance) from the embedding vector of diazepam in the latent space. We also interpolated between the structures of two common, but structurally unrelated, drugs vortioxetine and moclobemide, and presented molecules sampled along the interpolation line. The resulting molecular structures are presented in Figure S8.

### 3.4 Novelty and Diversity of Generated Molecules

**Figure 7:**
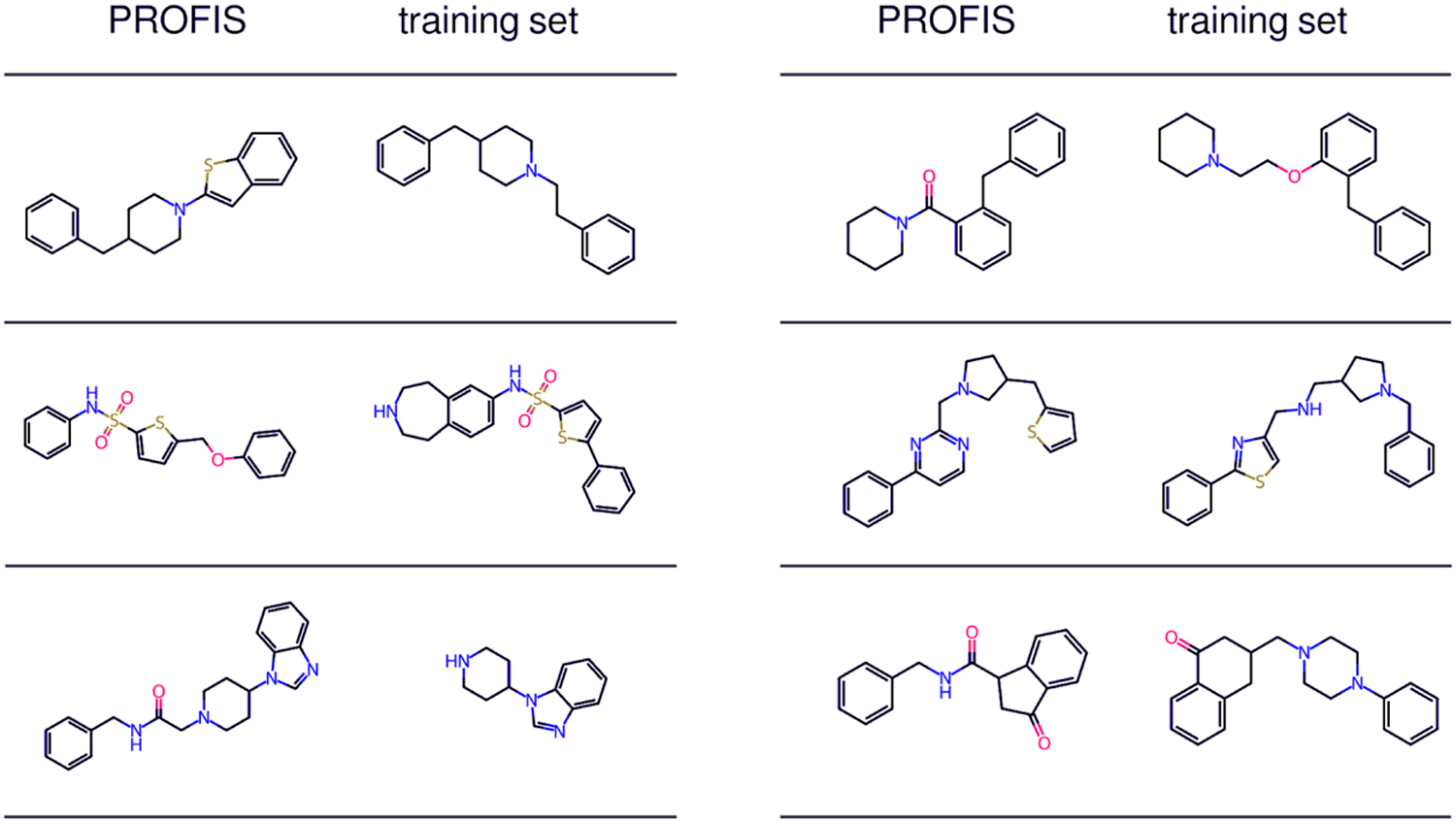
Scaffold-hopping potential of PROFIS as demonstrated on Bemis–Murcko scaffolds of molecules generated using PROFIS and the most similar scaffolds from the training D_2_R ligand dataset.

When using de novo drug design methods, researchers aim to explore structurally novel molecules in order to discover ligands with potential therapeutic properties. By examining the untapped chemical space, scientists can potentially identify novel drug candidates that exhibit unique pharmacological properties. Scaffold-hopping is one of the approaches to generate structurally novel molecules of desirable physicochemical and/or pharmacological properties, based on the structures of already-known compounds.^41^ It operates under the premise that compounds with distinct structural frameworks can exhibit similar biological activity, provided that they retain certain key features of the reference molecule. Figure 7 showcases the excellent scaffold-hopping potential of PROFIS by comparing pairs of Bemis–Murcko scaffolds, one generated by our model and its closest structural analog (based on Tanimoto distance) found in the D_2_R training set. The structures produced by PROFIS exemplify the outcome of a classical scaffold-hopping strategy applied to molecules from the D_2_R set.

**Figure 8:**
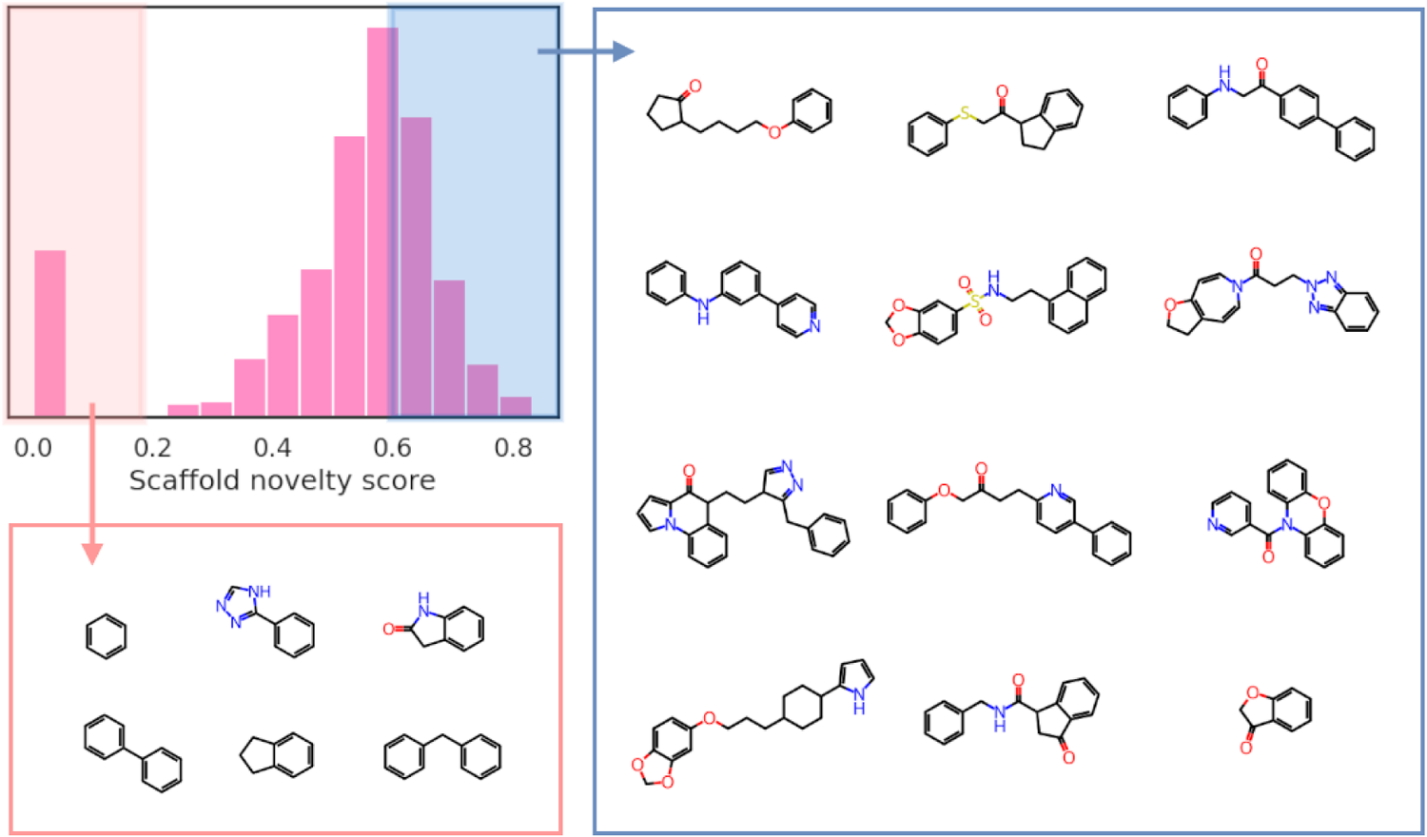
Distribution of scaffold novelty score for molecules generated by PROFIS (the smallest Tanimoto distance between the Bemis–Murcko scaffold of a generated molecule and all scaffolds present in the D_2_R ligand dataset). Most of the generated molecules are based on a novel scaffold. Some of the simpler molecules possess common scaffolds also present in the D_2_R ligand data set (benzene, biphenyl, 2-indolone, etc.).

To assess scaffold novelty, we calculated the Tanimoto distance between each scaffold produced by PROFIS and any scaffold within the entire D_2_R ligand training set, assigning a “scaffold novelty score” based on the smallest distance. Figure 8 illustrates the distribution of these scores alongside examples of the scaffolds. Notably, the majority of the molecules generated by PROFIS (∼90%) are based on novel scaffolds, with no similar scaffolds (smallest Tanimoto distance < 0.25) found in the training set. This finding further confirms that our generative model does not merely reproduce the scaffolds of D_2_R ligands encountered during training of the latent QSAR classifier; rather, the Bemis–Murcko scaffolds of the PROFIS-generated molecules are both novel and diverse. The high quality and structural novelty of the obtained molecules indicate their potential as future drug candidates, emphasizing the importance and value of PROFIS despite the relatively low number of valid structures generated in the output.

### 3.5 QSAR Applicability Domain

The concept of an applicability domain in QSAR modeling refers to identifying the specific chemical space where the model’s predictions are expected to be accurate and reliable.^42,43^ The unreliability of a classifier’s prediction may originate from two sources; either the modeled data point is distant from the training examples in the feature space or it is located close to the decision boundary of the classifier. The applicability domain is typically defined by employing some measure that assesses the reliability of individual predictions and can be based either on the distance to the training data (often termed *novelty detection*) or the distance to the decision boundary of a classifier (*confidence estimation)*.^43^ As the Bayesian search process of the PROFIS protocol is tasked with maximizing the confidence of classification, for our uses we will employ a distance-based applicability domain measure called SCAvg, which is a mean cosine similarity coefficient of query data point in the latent space to its three nearest training set neighbors.^44^ Such a metric is often called the *distance to model* (DM), and we will continue to use this term from now on.

**Figure 9:**
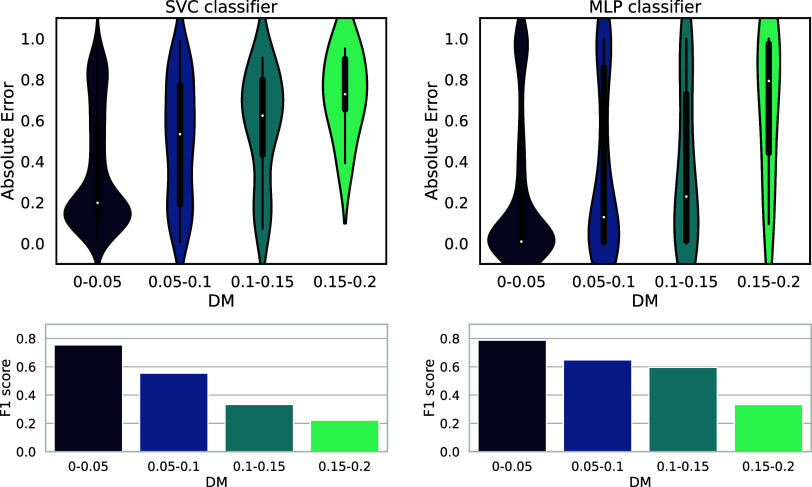
Defining the applicability domain of the trained QSAR models. The absolute error of classification and F1-score for different ranges of DM (distance to model, SCAvg measure).

Figure 9 shows the relation between the prediction error and DM. In this experiment, molecules were split into the training and validation subsets with a 9:1 ratio, following the same splitting technique as that described in Section 3.1. The testing molecules were binned into four DM ranges: below 0.05, between 0.05 and 0.1, between 0.1 and 0.15, and above 0.15. The top row shows the distribution of absolute errors (the distance of the model output to the correct class). We observe that the less similar the tested compound is to the training data, the greater the model’s error is on average. For compounds below 0.05 DM, the median absolute error is around 0.2 for SVC and 0.0 for MLP classifiers, so most predictions are correct. As for the MLP classifier, the median error is greater than 0.5 only for compounds with DM higher than 0.15. The SVC classifier seems not to be applicable to inputs with DM > 0.05. Those results suggest that our trained MLP QSAR model is able to output reliable predictions for fingerprint embeddings outside of the training set distribution in which SVC struggles. The bottom row of Figure 9 shows F1-scores in each range, which confirms that our predictor is more sensitive and precise for compounds similar to the training data. Based on these observations, we conclude that our MLP predictor is best applicable for compounds with DM less than 0.15, after which threshold the predictions start to degrade rapidly.

### 3.6 Molecular Docking

**Figure 10:**
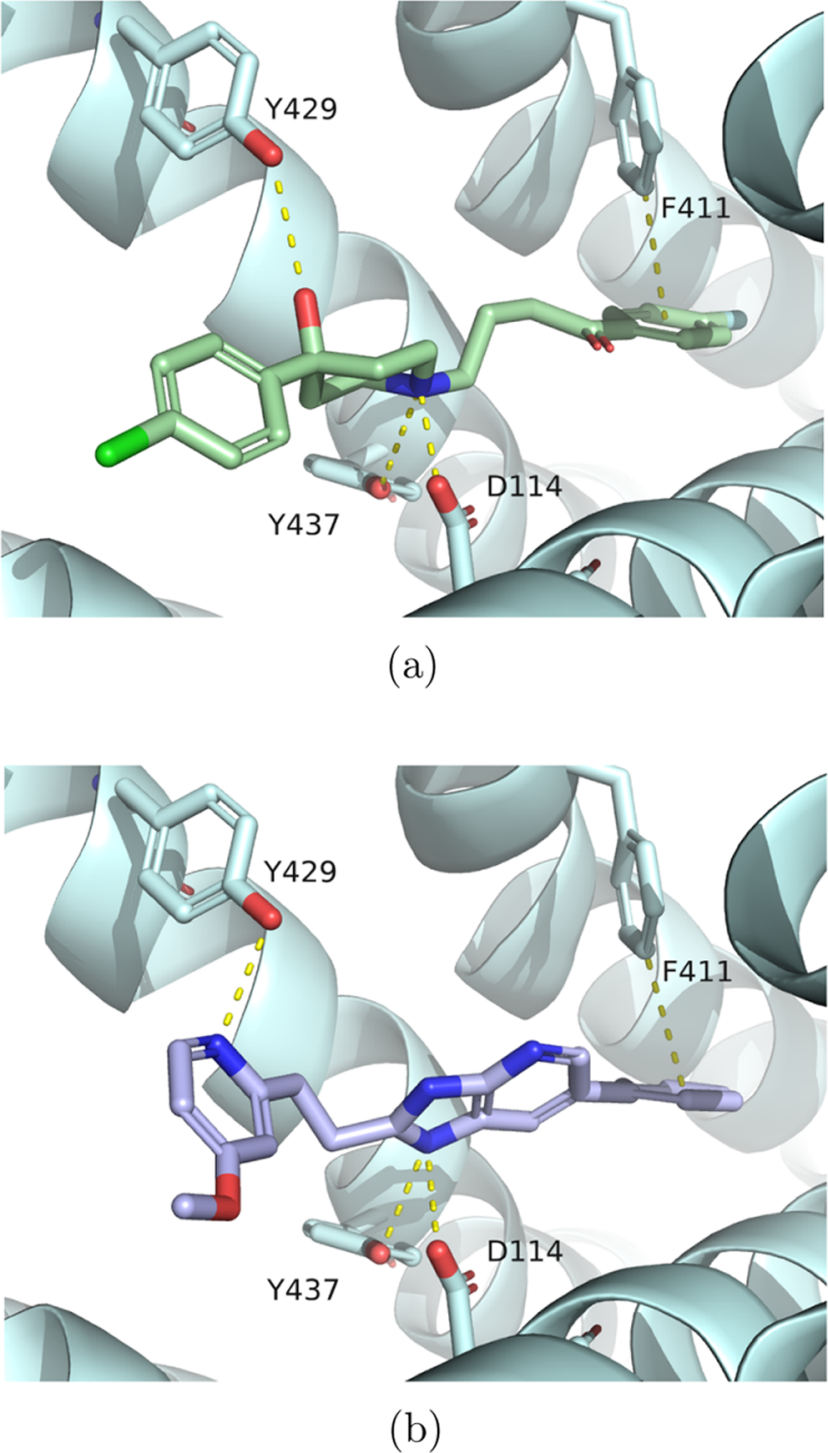
(a) Binding site occupancy of haloperidol, a potent D_2_R antagonist; (b) docking pose of compound 26, candidate D_2_R ligand generated by PROFIS. The ligand–protein interactions for the key residues are marked with dashed lines.

The D_2_R modulator activity of newly generated compounds was assessed by docking to the D_2_R crystal structure. A sample of molecules generated by PROFIS, in a number of 150, were selected by filtering the raw output of PROFIS by maximum ring size = 7, QED > 0.5, and clog *P*< 8. The filters were applied with the intent to remove highly lipophilic molecules and thus prevent the effects of GNINA default scoring function’s bias toward nonpolar molecules. Detailed analysis of the docking poses revealed an analogous binding pocket occupancy of known D_2_R binders and the generated molecules (Figure 10).

**Figure 11:**
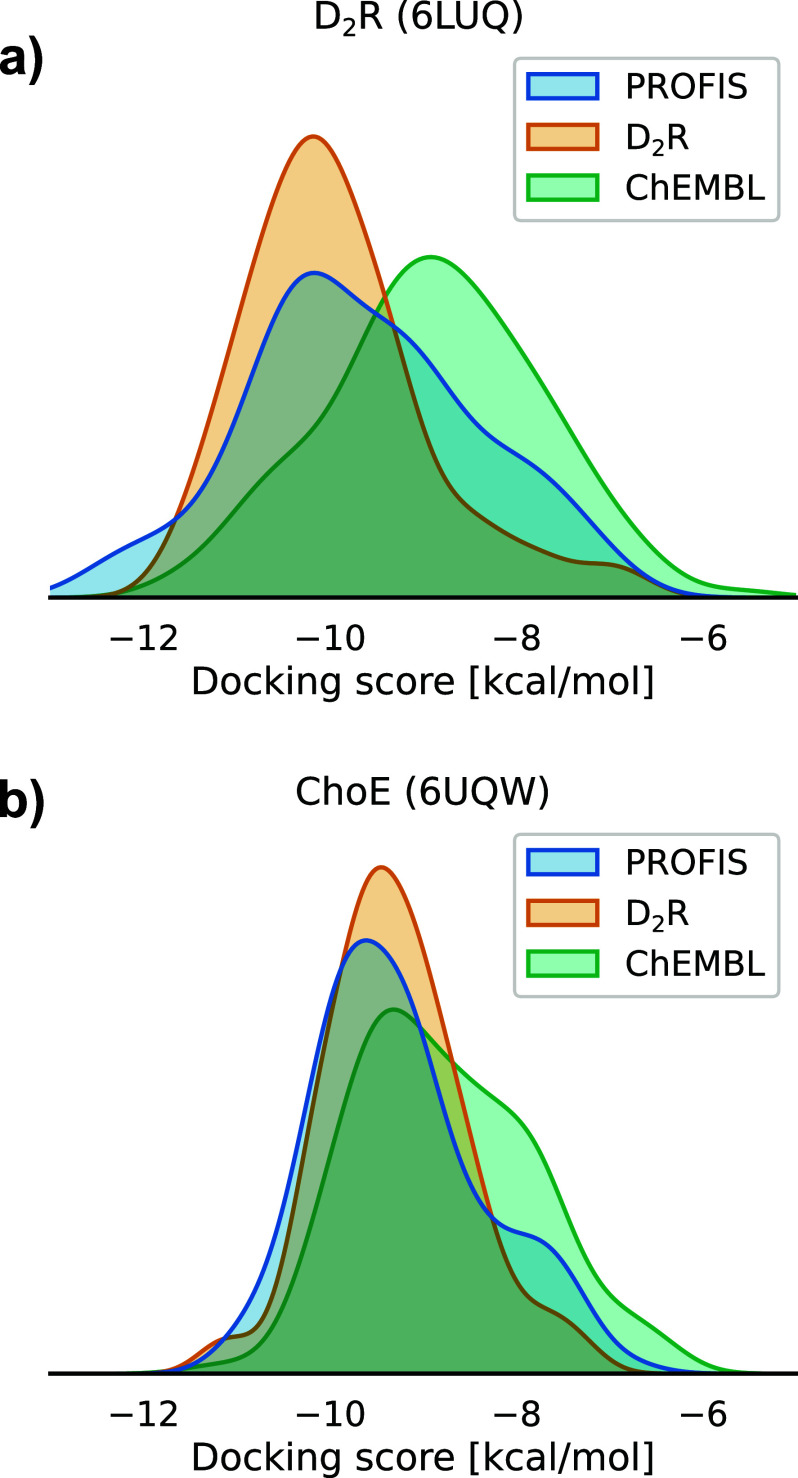
(a) D_2_R docking score distribution of PROFIS candidate ligands (blue) in comparison to known D_2_R binders (orange) and a sample of drug-like structures from the ChEMBL32 database (green); (b) analogous docking study results, but to an unrelated protein (bacterial acetylcholine esterase, ChoE). PDBIDs are provided in brackets.

The distribution of candidate ligands’ docking scores was compared with the docking results of a random sample of 150 drug-like molecules sourced from ChEMBL32. A comparison of molecular properties of the two sets is presented in Figure S7. As presented in Figure 11, the compounds generated by PROFIS exhibit stronger predicted D_2_R binding affinities (expressed by lower docking scores) than the random database sample. A Mann–Whitney *U* test was performed to verify whether the distribution of D_2_R docking scores obtained for PROFIS-generated compounds is indeed different from the distribution obtained for the ChEMBL32 sample. With a *p*-value of *p* < 0.00005, the null hypothesis of the distributions’ identity was rejected. Those observations stand as proof of our model’s capability to produce compound libraries of dedicated biological activity (target-focused libraries).

### 3.7 Analog Libraries Generation

**Figure 12:**
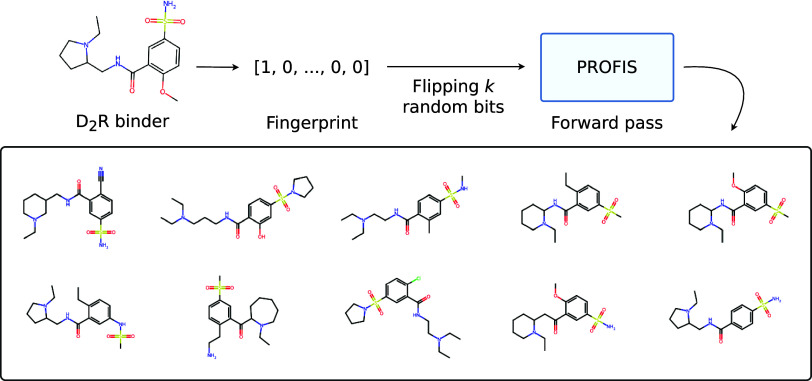
Examples of molecular structures obtained by introducing random noise (flipping *k* = 5 random bits) to the KRFP fingerprint of a D_2_R antagonist, sulpiride, and passing the resulting bit vectors through the whole PROFIS model. This approach yields libraries of diverse derivatives of the given compound that can then be screened in a typical lead optimization study.

Although the inference mode described in previous sections did not utilize our fingerprint encoder, it is important to note that this 3-layer fully connected network remains compatible with any bit vector input. Therefore, some artificial fingerprints can indeed be produced and passed through the trained PROFIS network, and the output molecules inspected in terms of their structure and properties. We hypothesized that introducing some random noise to FPs of known drug molecules at the input might yield many structurally related derivative compounds at the output. The following experiment was conducted: (1) First, the KRFP of the D_2_R antagonist drug, sulpiride, was calculated. (2) In 10,000 copies of this bit vector, five random bits were flipped to create a dataset of artificial fingerprints. These new fingerprints can be considered close neighbors of the original molecule in the 4860-dimensional KRFP space. (3) The noised fingerprints were then passed through PROFIS, resulting in 778 valid and unique derivatives of sulpiride. A sample of molecular structures obtained in this way is presented in Figure 12.

A computational hunt for new chemical structures that carry some form of resemblance to an already-known molecule resulted in a library of derivatives, which (after an optional filtering step) can be screened for activity using methods such as large-scale docking. This kind of approach proves to be useful in the process of lead optimization, as it automatizes the conception of new structural motifs or in designing new drugs around an existing patent claim.

**Figure 13:**
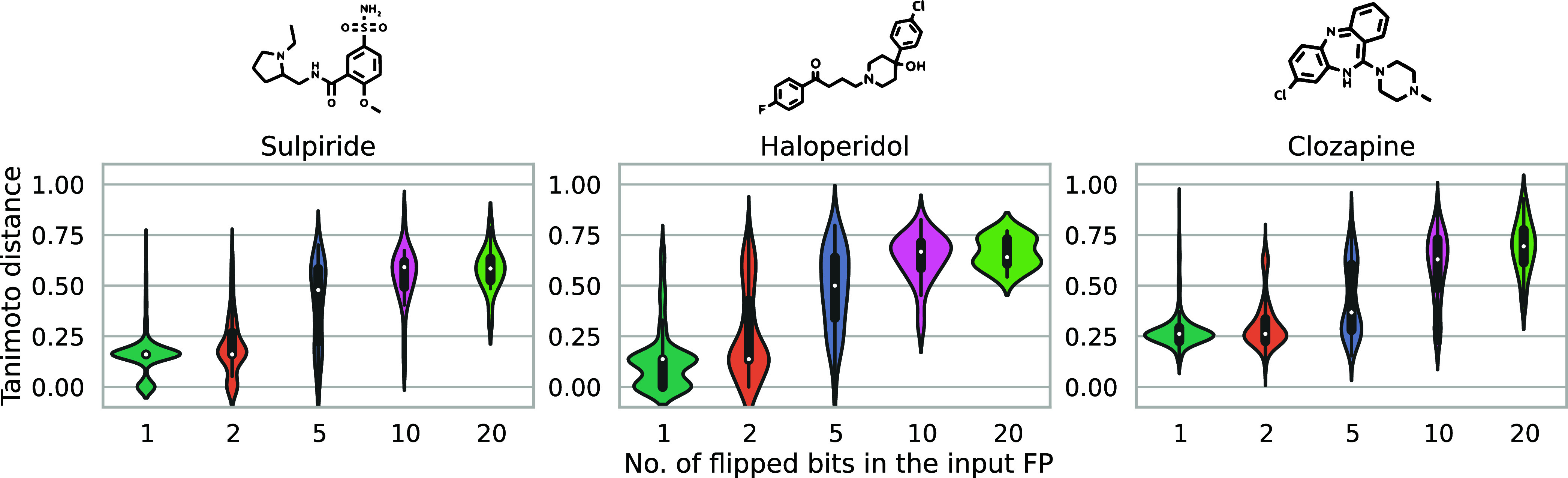
Tanimoto distance from the input to the output molecule positively correlates with the number of bits flipped in the input fingerprint (KRFP), as demonstrated on three drug molecules of a different molecular scaffold.

We used an example of three D_2_R binders of different structural classes—sulpiride, haloperidol, and clozapine—to understand how the amount of fingerprint noise affects the quality of output molecules (Figure 13). First, we generated five libraries of analogs for each of the drug molecules, with each library being based on fingerprints with a different number of randomly chosen bits having been flipped (1, 2, 5, 10, or 20). Then, based on KRFP fingerprints, we computed Tanimoto distance values for each pair of a newly generated analog and the original compound itself. We observed that the outputs become more structurally dissimilar to the input molecule with the increasing number of flipped input bits, which is not unexpected, given that our model did succeed in learning a meaningful, continuous mapping of molecular FPs onto SMILES strings.

**Figure 14:**
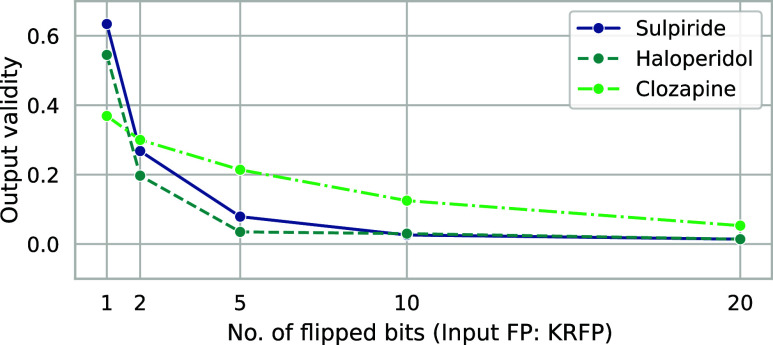
Validity of the output SMILES drops significantly for larger numbers of flipped input bits.

As demonstrated in Figure 14, the mean validity of the outputs declines rather rapidly with the increasing amount of noise introduced to the input fingerprints. Clearly, this behavior can be attributed to the inevitable emergence of bit combinations, which cannot be mapped to any realistic chemical structure. It is important to note that the rate of output validity decay may depend on the molecule of interest, as faux fingerprints based on clozapine KRFP were more resilient to SMILES syntax errors upon decoding, compared to the ones based on sulpiride and haloperidol KRFPs.

### 3.8 Limitations

An important consideration for applying our method is a moderately low validity of the output molecules, which increases the generation time by requiring more generation trials to be conducted. Representations such as SELFIES ensure 100% validity of the compounds, but the resulting structures produced by PROFIS with this notation are significantly less similar to typical drug-like compounds than when other representations are used. When generating SELFIES, we observed many undesirable artifacts, including heavily strained or unexpectedly large rings, potentially unstable or unrealistic atom connectivities, and long terminal alkane chains (see Figure S6). Those may be caused by how SELFIES reuses tokens to mark ring and branch sizes in order to ensure complete output validity. An especially common artifact produced by SELFIES-based PROFIS is improperly assembled aromatic systems and surprising cyclic polyenes, which are not present in molecules generated by the SMILES and DeepSMILES-based models. While the notation of (Deep)SMILES encodes aromaticity by using lowercase atomic symbols, SELFIES relies on alternating single or double bonds along the ring. As the electronic structure, and thus the chemical properties of aromatic systems, are not equal to those of conjugated polyenes, (Deep)SMILES’ convention of describing aromaticity seems more robust and may be one of the most important factors in the successful generation of realistic and drug-like organic molecules. We also noted that SELFIES-based sequential molecule generator often placed some kind of a three-atom ring at one end of the output molecule, which effectively corresponds to “[Ring1][Ring1]” being the last two tokens of a sequence. Taking those into consideration, we resort to representations that yield lower validity but can correctly capture the distribution of drug-like compounds. While employing DeepSMILES resulted in greater validity, the outputs often lacked structural complexity, which was only observed in the molecules generated with the SMILES-based PROFIS model.

It should be noted that the output validity of PROFIS is slightly compromised by employing a classifier-based Bayesian search in the latent space, which brings samples closer to the applicability domain boundary. Former studies employed an MLP that was attached to the latent space and trained along with the generator to provide higher validity.^16^ However, this is only possible with computed properties like clog *P* or QED. Activity datasets based on wet-lab experiments are too small to train useful generative models, and thus, other techniques leveraging unlabeled training data need to be used. By employing classifier-based optimization in the learned latent space, PROFIS enables the discovery of new potentially active compounds outside the distribution of already-known active compounds.

## 4 Conclusions

We present PROFIS, a novel approach to the generation of target-focused compound libraries by probing molecular FP space and decoding it into SMILES strings with RNNs. Its basis constitutes a VAE that transforms molecular FPs into a 32-dimensional latent space, which in turn can be searched for the representations of new potential ligands. The search for subspaces representing active compounds is carried out in conjunction with a QSAR model, which can be trained to discriminate between the latent representations of ligands and nonbinders for a chosen biological target. We show that the task of activity prediction is not compromised by embedding the molecular FPs in low-dimensional space. Decoding the latent vectors into SMILES strings with the use of a trained RNN enabled the generation of target-focused chemical libraries.

Compared with the other FP-to-SMILES models that aim to solve only the inverse mapping problem, our model can be used to optimize molecule activity by linking it to the substructures encoded in FPs. We show that our model displays excellent scaffold-hopping capabilities and that the structures generated by PROFIS are both novel and diverse. Moreover, we describe how our trained fingerprint encoder enables fast and reliable generation of analog libraries for a drug compound of interest, which may serve as a basis for lead optimization studies or the design of new drugs around an existing patent claim. We present the usefullness of our model by generating a library of candidate D_2_R ligands, as well as a library of structural analogs of an antipsychotic drug sulpiride. Nevertheless, the protocol can potentially be applied to any biological target and may be used by a wide community, thanks to the ready-to-use scripts provided by the authors.

We outline the issue of appropriate sequential encoding choice for molecular generative models, as our architecture worked well on SMILES, with the final compounds displaying molecular properties more favorable than those produced by DeepSMILES-based models. We found that SELFIES, despite its unique robustness, is not a suitable encoding format for the generation of realistic molecular structures under the PROFIS protocol. We discuss the impact of SELFIES ring closure and aromaticity syntax on the quality of the generated molecules. Those observations contribute to the broader discussion of the utility of SELFIES in generative ML tasks.

## Data Availability

**Data and Software Availability** The code is made available on GitHub under MIT license, along with all datasets used for training the model: https://github.com/hubertrybka/profis. Training sets in SMILES format and lists of generated compounds in SMILES format are available in the Supporting Information.

## Supporting Information Available

Molecular properties of the D_2_R ligands set, which was used to train the latent space classifier (Figure S1); raw output of a model trained on ECFP4 fingerprints with SMILES as the output format, an MLP QSAR model and Bayesian search *bounds* = 2 were used during inference (Figure S2); raw output of a model trained on KRFP fingerprints with SMILES as the output format, an MLP QSAR model and Bayesian search *bounds* = 2 were used during inference (Figure S3); distribution of molecular mass for compound libraries produced by ECFP4 and KRFP-based PROFIS models at different *bounds* parameter of the Bayesian latent space search (Figure S4); raw output of PROFIS trained on KRFP fingerprints with DeepSMILES as the output format, although the use of DeepSMILES ensures higher validity of generated strings than in the case of SMILES, the overall quality of generated libraries suffers from the lack of structural complexity and prevalence of long, lipophilic carbon chains (Figure S5); raw output of PROFIS trained on KRFP fingerprints with SELFIES as the output format, the common decoding artifacts include either heavily strained or unexpectedly large rings, malformed aromatic systems, and long carbon chains (Figure S6); molecular properties of the docked PROFIS output molecules (blue) and a reference random sample of drug-like compounds from the ChEMBL32 database (orange) (Figure S7); latent space continuity showcase experiment, (left) examples of molecular structures decoded from latent vectors at a given euclidean distance form the embedding of diazepam, (right) interpolation of vortioxetine, and moclobemide embeddings, with the example structure sampled along the interpolation line (Figure S8); hyperparameter values used for training the best-performing VAE models (Table S1); hyperparameter grid used for VAE hyperparameter tuning (Table S2); hyperparameter grids used for tuning latent space classifiers (Table S3); and list of tokens selected as the alphabet of SMILES, SELFIES, and DeepSMILES sequential generators (Table S4) (PDF)
RNN training data set composed of ChEMBL32 and ZINC compounds in SMILES format (File S1) (TXT)
Dataset of D_2_R ligands used to train the latent space classifier (File S2) (TXT)
Unfiltered output SMILES of an ECFP4-based PROFIS tasked with the generation of potential D_2_R binders (File S3) (TXT)
Unfiltered output SMILES of a KRFP-based PROFIS tasked with the generation of potential D_2_R binders (File S4) (TXT)
Library of diverse sulpiride analogues and derivatives, generated by passing noised sulpiride KRFPs through the PROFIS model (File S5) (TXT)
