# Supplementary material for: PROFIS: Design of Target-Focused Libraries by Probing Continuous Fingerprint Space with Recurrent Neural Networks
Source: J Chem Inf Model. 2025 Apr 28;65(9):4412–25. doi: 10.1021/acs.jcim.5c00698 (PMC12076512; doi:10.1021/acs.jcim.5c00698)
Supplement: Supplementary file 1 — ci5c00698_si_001.pdf [file ci5c00698_si_001.pdf]

Supporting Information for
PROFIS: Design of target-focused libraries by probing continuous fingerprint space with recurrent neural networks

Hubert Rybka,[†,‡] Tomasz Danel,[*,‡,§] and Sabina Podlewska[*,¶,§]

†*Doctoral School of Exact and Natural Sciences, Jagiellonian University, Łojasiewicza 11, 30-348, Kraków, Poland*

‡*Faculty of Chemistry, Jagiellonian University, Gronostajowa 2, 30-387 Kraków, Poland*

¶*Maj Institute of Pharmacology, Polish Academy of Sciences, Smętna 12, 31-343 Kraków, Poland*

§*Contributed equally*

E-mail: tomasz.danel@uj.edu.pl; smusz@if-pan.krakow.pl

Practical considerations

The generative capacity of PROFIS can be utilized with any biological target, as long as a dataset of known ligands with their biological activities (either numerical or categorical) is available. As the predictive power of a classifier is dependent on the size of its training set, one should be wary when interpreting the results of running PROFIS on small ligand sets. One could adopt a rule of thumb that assumes providing no less than 5 training examples per feature[1], which would result in a dataset of minimum $32 * 5 = 160$ compounds.

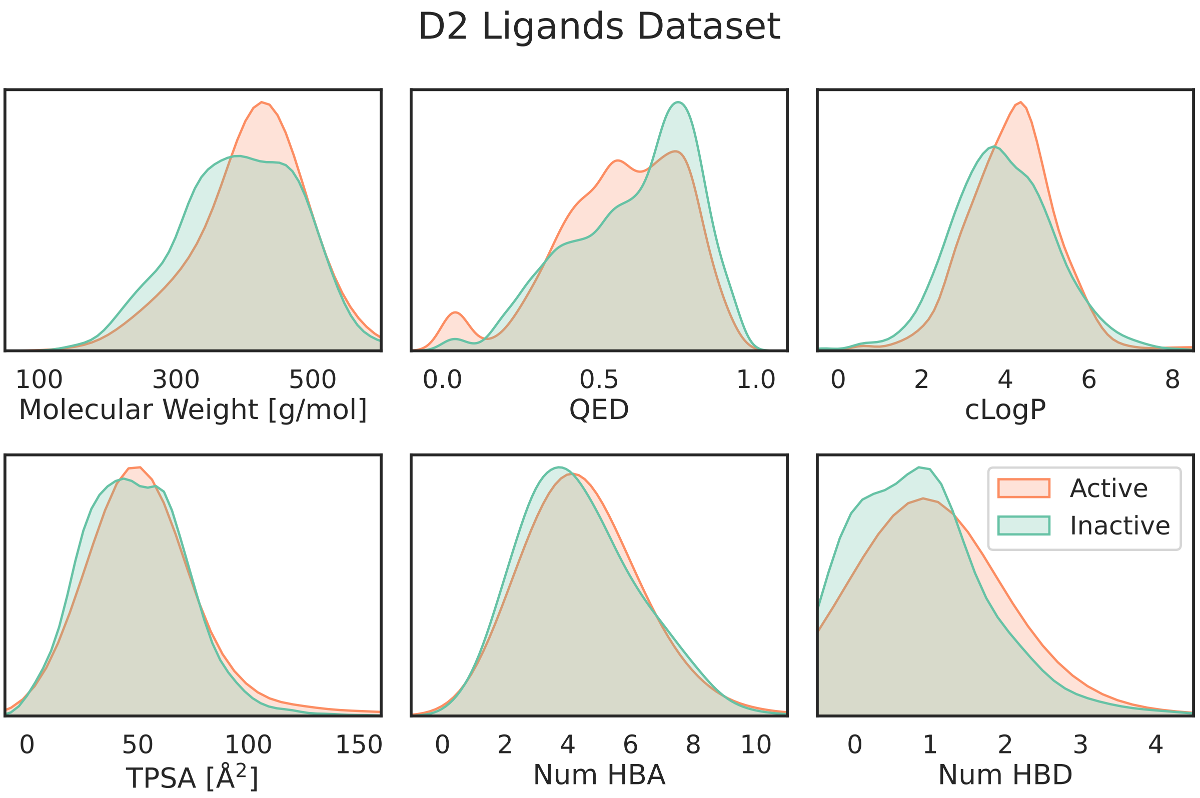

Figure S1: Molecular properties of the D_2R ligands set, which was used to train the latent space classifier.

The process of retraining a latent classifier for a new biological target is not resource-heavy and can be executed on a consumer-grade machine. For all calculations, we used a PC equipped with AMD Ryzen 9 7900X (24 CPU threads) and 64 GiB of memory. Training a latent space QSAR model with set hyperparameters on such a machine takes up to a minute, and up to 20 minutes when optimizing hyperparameters with a double-nested CV strategy. We also tried to train the classifier models on a more affordable computer, namely a 2018 Lenovo ThinkPad T480 with 8 CPU cores. Training with set hyperparameters took no longer than 2 minutes for the D_2R ligands dataset described in our paper, and up to an hour with hyperparameter optimization.

Retraining VAE is not necessary, as the network weights for models trained on both KRFP and ECFP4 fingerprints are available to be downloaded from an online repository. The training of VAE must realistically be carried out on a GPU-equipped machine and takes

24 hours to converge on NVIDIA RTX 4090.

Bayesian latent space search is probably the most time-consuming step of the PROFIS protocol. On our 24-thread CPU it takes about 20 minutes to identify 1,000 latent vectors corresponding to molecules of high predicted activity. Executing this step on the 8-thread CPU of the aforementioned Thinkpad laptop takes approximately five times as long. The search script is designed to be run on multi-core processors and greatly benefits from parallelization.

Decoding artifacts

In Figures S5 and S6, we show examples of molecules generated by DeepSMILES and SELFIES-based RNNs, which include a variety of extremely lipophilic, chemically unrealistic, or potentially unstable molecules.

Table S1: Hyperparameter values used for ECFP4 and KRFP models training.

Variable	Value	Comment
batch_size	256	-
epochs	1000	-
learn_rate	0.0002	-
kld_weight	0.1	value of β in the model's objective function formula
encoding_size	32	dimensionality of the encoded fingerprint space
hidden_size	512	size of a GRU cell hidden state
num_layers	2	number of GRU layers
dropout	0	rate of dropout applied after each but last GRU layer
fc1_size	1024	FP encoder's 1st fully-connected layer size
fc2_size	1024	FP encoder's 2nd fully-connected layer size
annealing_max_epoch	50	the epoch on which KLD annealing ends
annealing_shape	cosine	KLD annealing shape
teacher_ratio	0.2	teacher forcing ratio

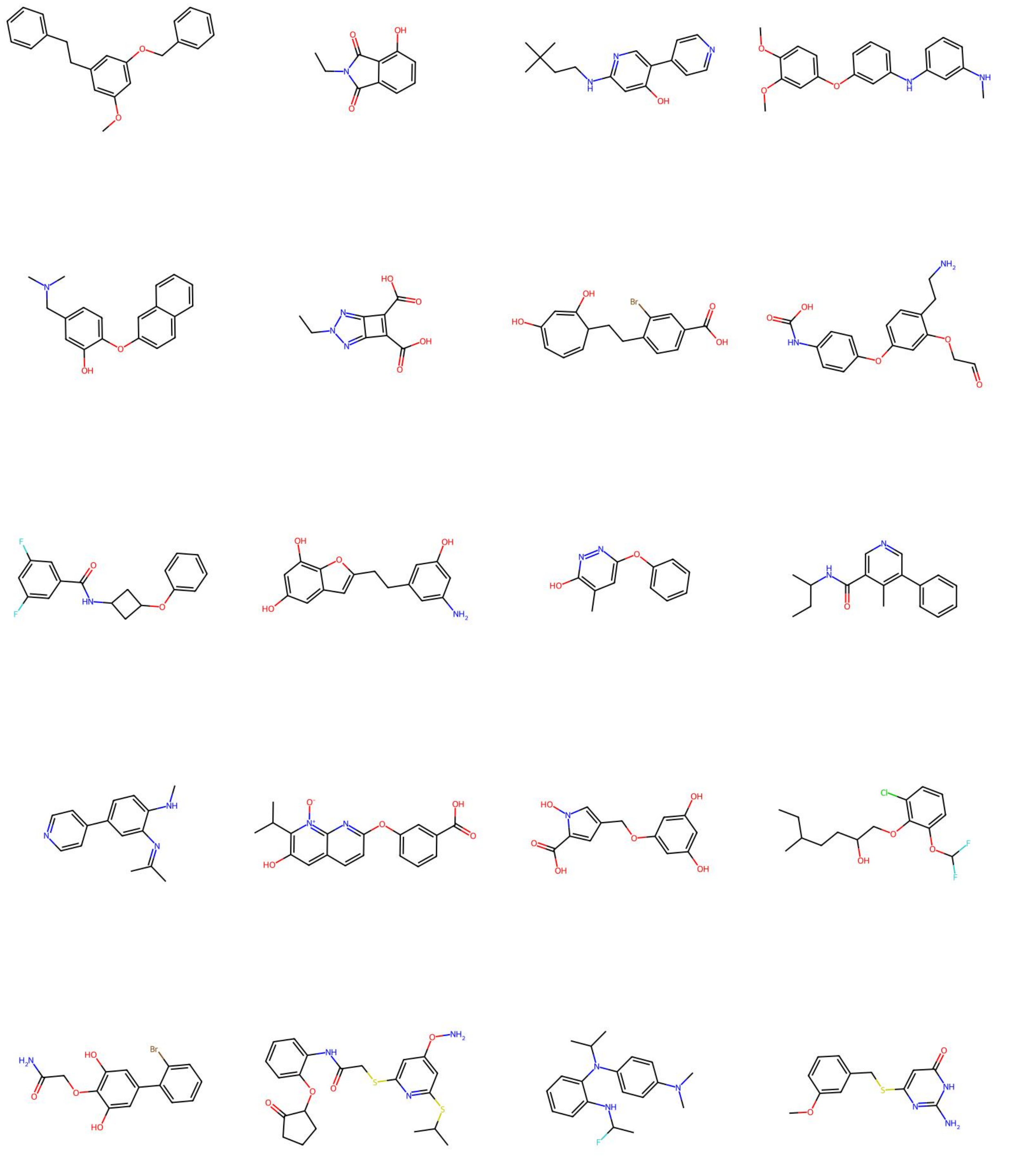

Figure S2: Raw output of PROFIS trained on ECFP4 fingerprints with SMILES as the output format. An MLP QSAR model and Bayesian search *bounds* = 2 were used during inference.

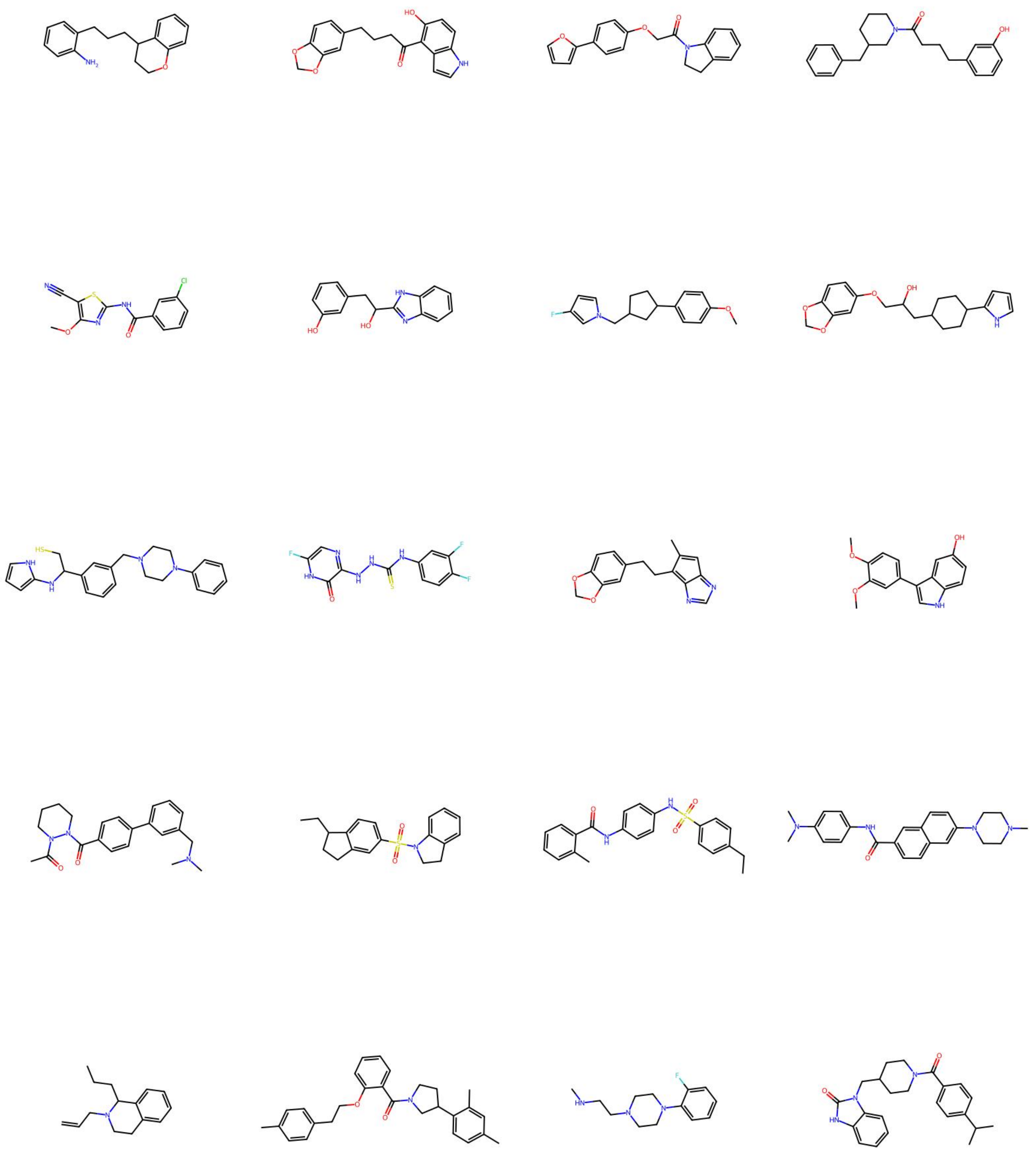

Figure S3: Raw output of PROFIS trained on KRFP fingerprints with SMILES as the output format. An MLP QSAR model and Bayesian search *bounds* = 2 were used during inference.

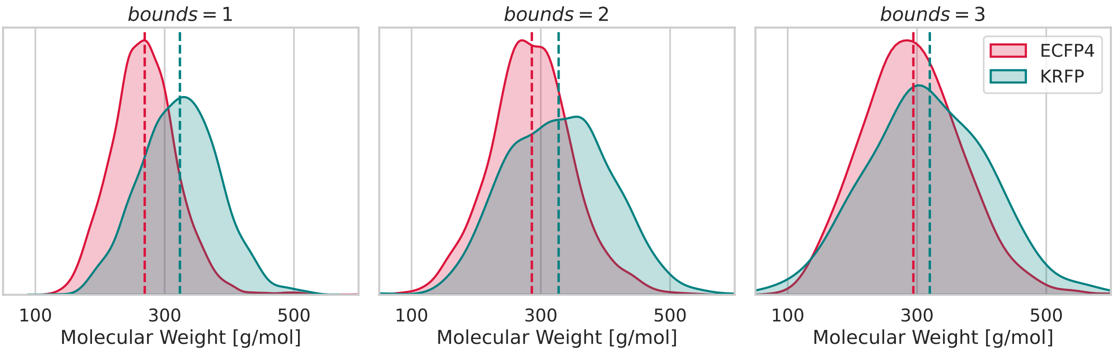

Figure S4: Distribution of molecular mass for compound libraries produced by ECFP4 and KRFP-based PROFIS models at different *bounds* parameter of the Bayesian latent space search.

Table S2: Hyperparameter grid used for VAE hyperparameter tuning

Hyperparameter	Value
hidden_size	[512, 1024, 2048]
num_layers	[1, 2, 3]
dropout	[0, 0.1, 0.3]
kld_weight	[0.01, 0.05, 0.1]
teacher_ratio	[0, 0.2, 0.5, 0.9]
fc1_size	[1024, 2048]
fc2_size	[512, 1024]
fc3_enabled	[True, False]
fc3_size	[256, 512]

Latent space classifier

The latent classifier was trained on the dataset of D_2R ligands extracted from ChEMBL. The properties of compounds in this dataset are summarized in Figure S1. These ligands are small molecules with a weight between 200 and 600 g/mol. Most of the molecules contain one hydrogen bond donor (HBD) and on average four hydrogen bond acceptors (HBA). Most compounds are in the drug-like logP range, with only a few exceptions with logP > 5.

A nested stratified five-fold cross-validation strategy was used to tune the hyperparameters and evaluate the performance of different latent space classifiers simultaneously. The CV strategy was implemented as follows:

1) The dataset was split into 5 parts, with one of the parts designated as the test set

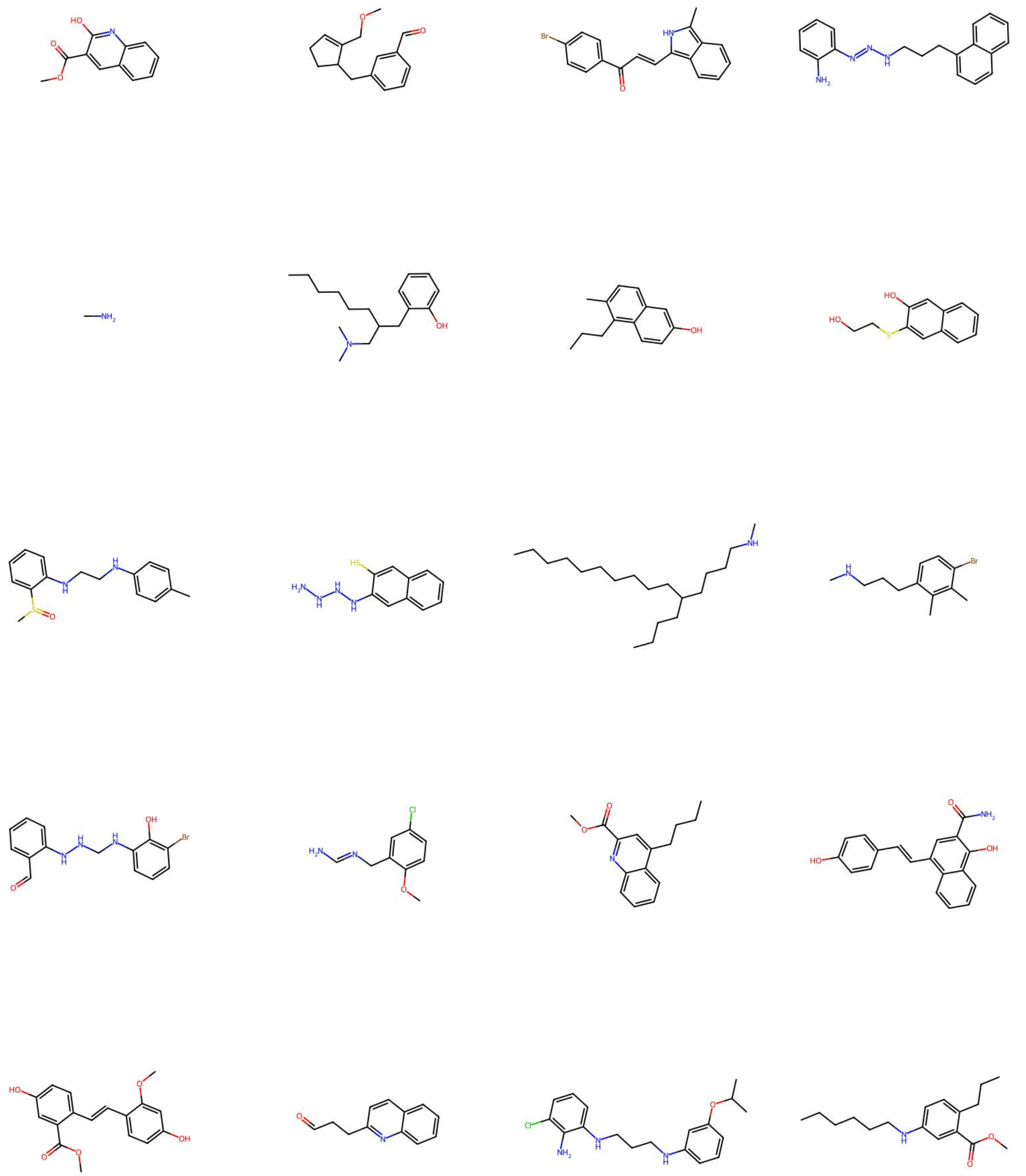

Figure S5: Raw output of a model trained on KRFP fingerprints with DeepSMILES as the output format. Although the use of DeepSMILES ensures higher validity of generated strings than in the case of SMILES, the overall quality of generated libraries suffers from the lack of structural complexity and prevalence of long, lipophilic carbon chains.

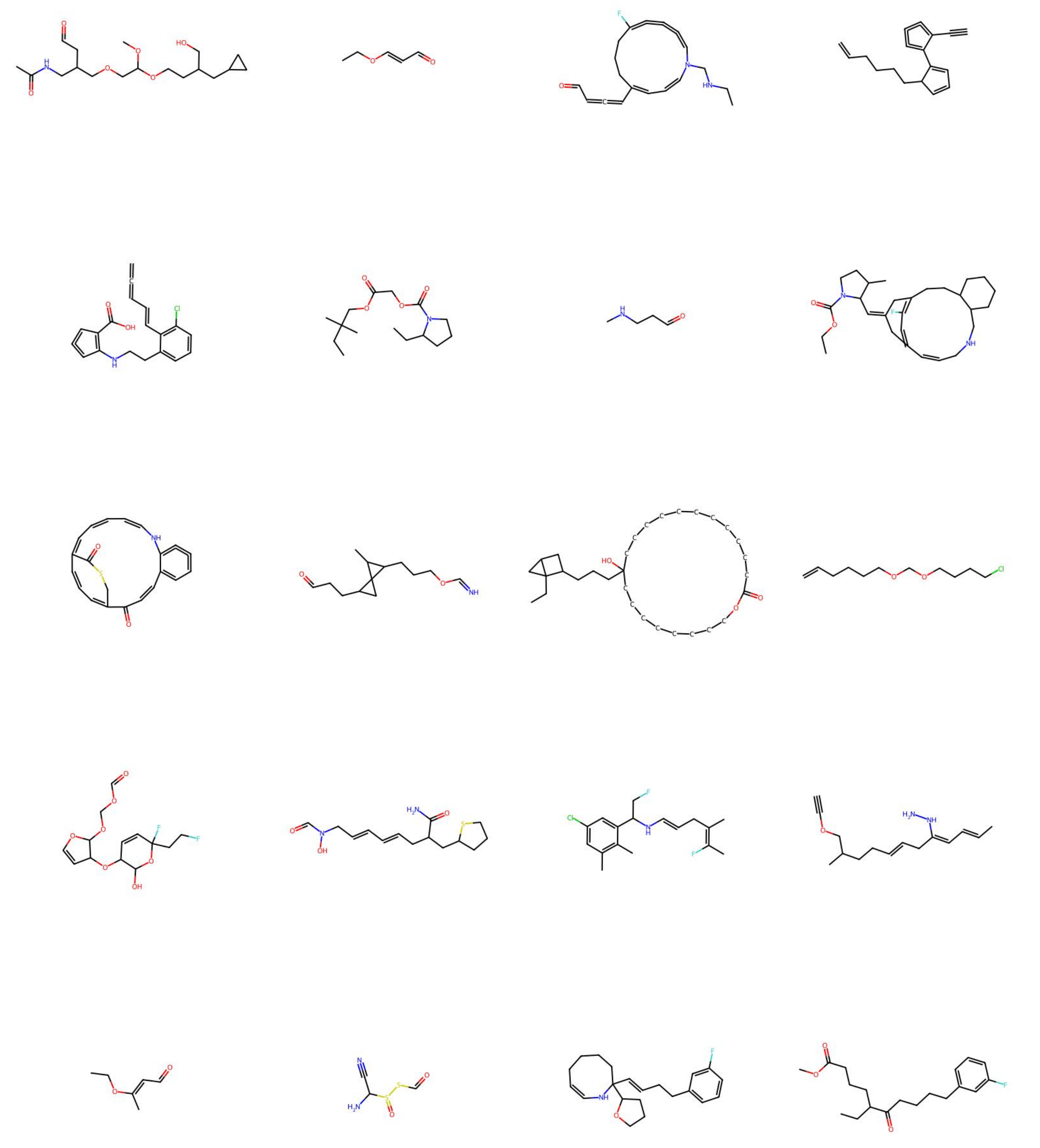

Figure S6: Raw output of a model trained on KRFP fingerprints with SELFIES as the output format. The common decoding artifacts include; either heavily strained or unexpectedly large rings, malformed aromatic systems, and long carbon chains.

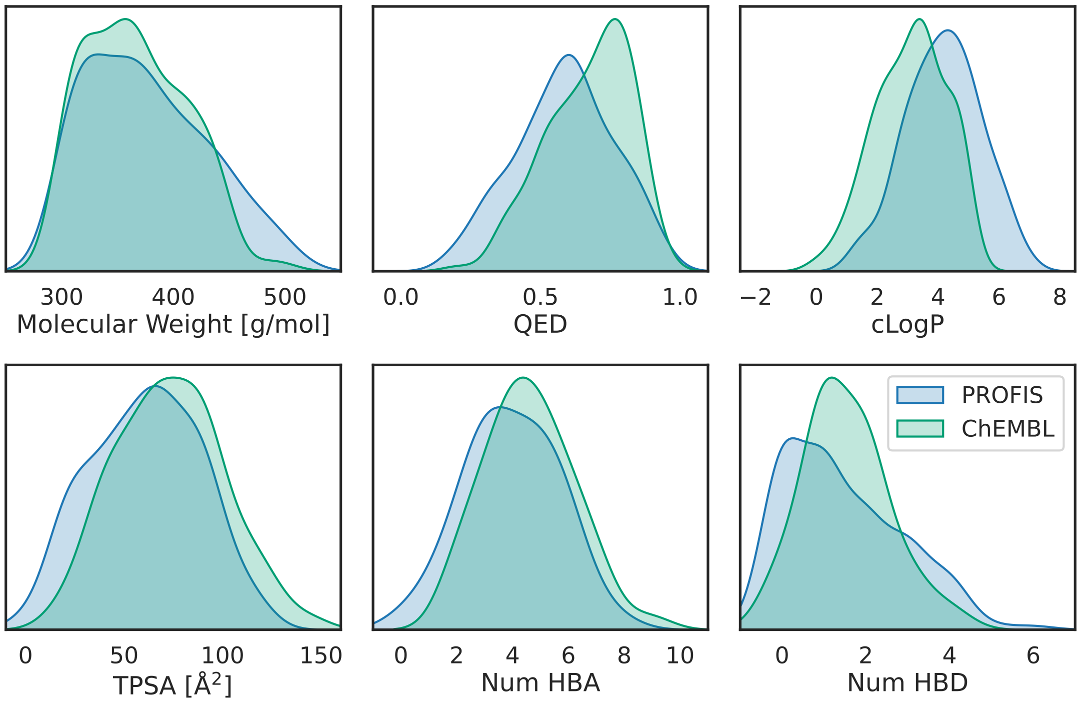

Figure S7: Molecular properties of the docked PROFIS output molecules (blue) and a reference random sample of druglike compounds from the ChEMBL32 database (orange).

for the final performance evaluation. The remaining 4 parts were combined into a single training set. This is the outer loop of the nested CV.

2) This training set was, again, partitioned five-fold. The model was trained on 4 of the parts and evaluated on the remaining one. This is the inner loop of the nested CV, which was repeated 5 times. The best hyperparameter combination is selected based on mean ROC AUC score on the evaluation subsets.

4) The performance of the best model is evaluated on the previously designated testing set. This is a part of the outer loop, which is repeated 5 times, yielding 5 values of accuracy and ROC AUC metrics, for which the mean and standard deviation values are calculated and reported.

Table S3: Hyperparameter grids used for tuning latent space classifiers. Best-performing values are marked in bold for ECFP4-based latent space and underlined for KRFP-based latent space.

SVM

Hyperparameter	
kernel	[**'rbf'**]
C	[0.1, 1, **10**, 100, 500]
gamma	[0.001, 0.0001, **'scale'**]
kernel	['linear']
C	[0.1, 1, 10, 100]

RF

Hyperparameter	Value
n_estimators	[100, 250, **500**, 1000]
max_features	[**'sqrt'**, 'log2', None]
max_depth	[3, **6**, 9, None]
max_leaf_nodes	[6, 9, 12, **18**]

XGB

Hyperparameter	Value
n_estimators	[50, 100, **250**, 500]
min_child_weight	[3, **6**, 9, 12]
max_depth	[9, 12, 18, **24**]
gamma	[**0**, 0.1, 0.2]
subsample	[0.6, **0.8**, 1.0]

MLP

Hyperparameter	Value
hidden_layer_sizes	[[16], [32], [64], [128], [256], [512], **[1024]**]
learning_rate_init	[**0.001**, 0.0001, 0.00001]
alpha	[**0**, 0.000]
hidden_layer_sizes	[[16, 8], [32, 16], [64, 32], [128, 64], [256, 128], [512, 256]]
learning_rate_init	[0.001, 0.0001, 0.00001]
alpha	[0, 0.0001]

Table S4: List of tokens selected as the alphabet of SMILES, SELFIES, and DeepSMILES sequential generators

SMILES	SELFIES	DeepSMILES	
C	[#Branch1]	C	3
c	[#Branch2]	c	4
(	[#C]	)	5
)	[#N]	n	6
n	[=Branch1]	N	7
N	[=Branch2]	-	8
-	[=C]	=	9
=	[=N+1]	O	%10
O	[=N]	o	%11
o	[=O]	[nH]	%12
[nH]	[=Ring1]	s	%13
s	[=Ring2]	S	%14
S	[=S]	F	%15
F	[Br]	Cl	%16
Cl	[Branch1]	Br	%17
Br	[Branch2]	I	%18
#	[C]	P	%19
[N+]	[Cl]	#	%20
[n+]	[F]	[N+]	%21
P	[I]	[n+]	%22
I	[N+1]		%23
1	[NH1]		%24
2	[N]		%25
3	[O]		%26
4	[P]		%27
5	[Ring1]		%28
6	[Ring2]		
7	[S]		
8			

Comparison to smiles2smiles VAEs

To draw a fair comparison between PROFIS and a more straightforward molecular VAE architecture, such as smiles2smiles, we trained a model similiar to Gómez-Bombarelli's molecular VAE described in their 2018 paper[2] on our dataset of 1,126,085 druglike compounds. We based our code on a specific PyTorch implementation of said network from Akshay Subramanian's GitHub repository.[3]

The smiles2smiles model outperforms PROFIS in its ability to recreate valid SMILES from the input data. On a holdout validation set of druglike molecules, the mean output validity of a trained smiles2smiles is in range of 50-60%, while PROFIS does not produce more than 40% (ECFP4) or 30% (KRFP) valid SMILES strings on the same validation set.

On the other hand, the trained smiles2smiles VAE performs slightly worse as a generative model than PROFIS does. Decoding random samples drawn from the prior distribution yields on average only 3.8% of correct SMILES, which is actually lower than 6.8% validity reported by us for ECFP4-based PROFIS and 5.7% for KRFP-based PROFIS.

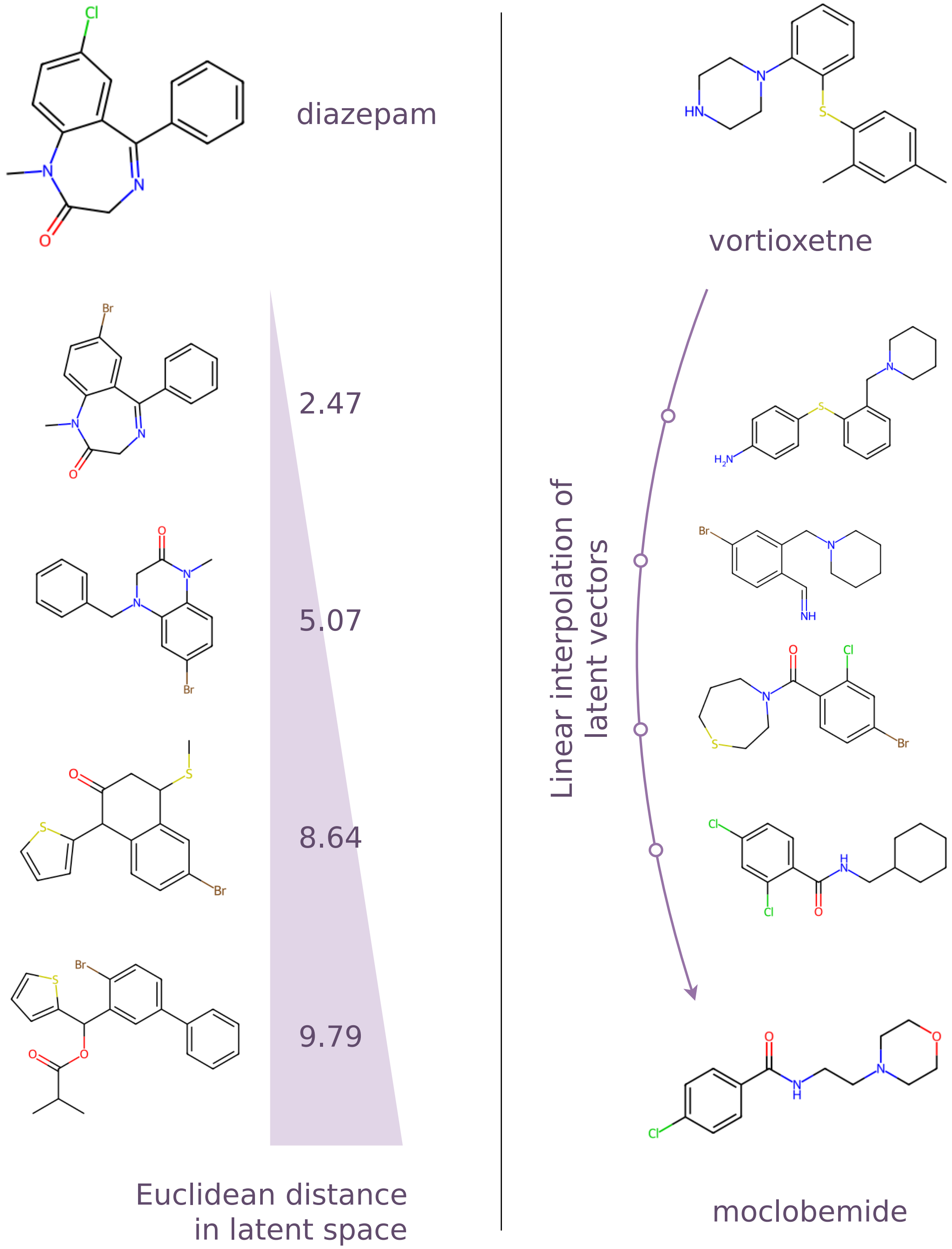

Figure S8: Latent space continuity showcase experiment. (left) Examples of molecular structures decoded from latent vectors at given euclidean distance form the embedding of diazepam. (right) Interpolation of vortioxetine and moclobemide embeddings, with example structure

References

(1) Theodoridis, S.; Koutroumbas, K. *Pattern recognition*; Elsevier, 2006.

(2) Gómez-Bombarelli, R.; Wei, J. N.; Duvenaud, D.; Hernández-Lobato, J. M.; Sánchez-Lengeling, B.; Sheberla, D.; Aguilera-Iparraguirre, J.; Hirzel, T. D.; Adams, R. P.; Aspuru-Guzik, A. Automatic chemical design using a data-driven continuous representation of molecules. *ACS central science* **2018**, *4*, 268–276.

(3) Subramanian, A. molecular-vae. `https://github.com/aksub99/molecular-vae`, 2019.
